# Secondary Metabolites from *Artemisia* Genus as Biopesticides and Innovative Nano-Based Application Strategies

**DOI:** 10.3390/molecules26103061

**Published:** 2021-05-20

**Authors:** Bianca Ivănescu, Ana Flavia Burlec, Florina Crivoi, Crăița Roșu, Andreia Corciovă

**Affiliations:** 1 Department of Pharmaceutical Botany, Faculty of Pharmacy, “Grigore T. Popa” University of Medicine and Pharmacy, 16 University Street, 700115 Iasi, Romania; bianca.ivanescu@umfiasi.ro; 2 Department of Drug Analysis, Faculty of Pharmacy, “Grigore T. Popa” University of Medicine and Pharmacy, 16 University Street, 700115 Iasi, Romania; acorciova@yahoo.com; 3 Department of Pharmaceutical Physics, Faculty of Pharmacy, “Grigore T. Popa” University of Medicine and Pharmacy, 16 University Street, 700115 Iasi, Romania; 4 Department of Experimental and Applied Biology, Institute of Biological Research Iasi, 47 Lascăr Catargi Street, 700107 Iasi, Romania; craita2002@yahoo.com

**Keywords:** antifungal, antibacterial, insecticidal, nematicidal, phytotoxic, herbicidal, non-target organism, nanoparticles, nanoemulsions

## Abstract

The *Artemisia* genus includes a large number of species with worldwide distribution and diverse chemical composition. The secondary metabolites of *Artemisia* species have numerous applications in the health, cosmetics, and food sectors. Moreover, many compounds of this genus are known for their antimicrobial, insecticidal, parasiticidal, and phytotoxic properties, which recommend them as possible biological control agents against plant pests. This paper aims to evaluate the latest available information related to the pesticidal properties of *Artemisia* compounds and extracts and their potential use in crop protection. Another aspect discussed in this review is the use of nanotechnology as a valuable trend for obtaining pesticides. Nanoparticles, nanoemulsions, and nanocapsules represent a more efficient method of biopesticide delivery with increased stability and potency, reduced toxicity, and extended duration of action. Given the negative impact of synthetic pesticides on human health and on the environment, *Artemisia*-derived biopesticides and their nanoformulations emerge as promising ecofriendly alternatives to pest management.

## 1. Introduction

The *Artemisia* L. genus contains over 500 species, herbaceous plants and shrubs, widespread in the northern hemisphere, in Asia, Europe, and North America. *Artemisia* species are found in various ecosystems, ranging from arid regions to wetland at sea level as well as in the mountains. The largest number of species are located in the steppes of Asia [1]. Common names of *Artemisia* species are wormwood, mugwort, and sagebrush. Due to their biological and chemical diversity, *Artemisia* species have numerous applications in the treatment of plant and human diseases, in cosmetic and pharmaceutical industry. In addition, various *Artemisia* species are used all over the world as foods, spices, condiments, and beverages [2]. Many important medicinal plants belong to this genus and exert a range of therapeutic actions: antibacterial, antifungal, antiviral, antiprotozoal, anthelmintic, anti-inflammatory, anti-ulcer, appetite stimulating, hepatoprotective, antispasmodic, bronchodilator, hypolipidemic, antihypertensive, analgesic, neuroprotective, neurotrophic, anti-depressant, antioxidant, cytotoxic, antitumor, estrogenic, anti-allergic, immunomodulatory, insecticidal, repellent, and anticonvulsant [3,4,5,6,7,8].

Most *Artemisia* species are aromatic plants that produce volatile oil in the secretory hairs on the aerial organs but also through the secretory ducts in the parenchyma tissues. Essential oils could be used as biocontrol agents based on the antibacterial, antifungal, repellent, insecticidal, nematicidal, and phytotoxic effect of volatile compounds. Moreover, the complex mixture of substances with different mechanisms of action, often having synergistic activity, can be effective in preventing the emergence of resistant strains of phytopathogens [9,10,11,12].

The global use of synthetic pesticides has many disadvantages, such as high cost, danger to non-target organisms, accumulation of pesticide residues in the environment, the emergence of resistant phytopathogenic strains, and negative impact on human health [12]. In contrast, biological pesticides can achieve pest management in an environmentally friendly way and could become safer alternatives for the treatment of crop diseases. Many agents are considered biopesticides, such as viruses, microbes, fungi, entomophagous invertebrates, parasitoids, predators, and substances produced by living organisms such as bacteria, fungi, plants, algae, animals, etc. Throughout this review, we will use the word “biopesticides” for plant-derived substances or extracts. During evolution, plants developed different mechanisms to defend themselves from predators and diseases by producing substances with bactericidal, fungicidal, insecticidal, nematicidal, or repellent activity. At present, these phytochemicals are explored as biocontrol agents for crops integrated pest management. Plant compounds are cheaper, safer for farmers, less toxic to non-target organisms, and rapidly degraded in the environment [13].

In this context, numerous researchers have identified new potential biopesticides in plants of the *Artemisia* genus. Since most species are fragrant, the vast majority of investigations have focused on the biological actions of volatile oils and compounds. Essential oils contain a variety of volatile molecules such as mono- and sesquiterpenes as well as phenolic-derived aromatic and aliphatic components [1]. The percentage of individual compounds in the essential oil is variable and depends on genetic factors (species, chemotype), plant origin, plant organ, period of harvest or developmental stage, environmental factors (climate, altitude, sun exposure), and cultivation conditions. Qualitative and quantitative differences in the composition of the essential oil can also be caused by drying methods, extraction procedure and time, quantification methods, and conditions of analysis [11]. All these elements could change the chemical composition of an essential oil, leading to changes in activity; thus, standardization is necessary to guarantee the effect, and also for regulatory and marketing purposes. Moreover, plants with desirable pesticide action may give low yields of essential oil, hence the need for new and more efficient extraction methods, which will increase the quantity and quality of extracted oil while reducing the time and cost of extraction [14].

This review focuses on significant and recent data related to the secondary metabolites’ activity of *Artemisia* species against plant pests and to the appropriate formulation and application of these biopesticides. The review has been assembled using references from major databases such as PubMed, Science Direct, Scopus, SpringerLink, Google Scholar, and Web of Science. There is an abundance of papers that evaluate the pesticide activity of *Artemisia* species in vitro, but only a handful include *in planta* or greenhouse experiments, and even fewer contain field tests. Furthermore, there is a shortage of studies regarding the effect on non-target organisms. Since various compounds and extracts, especially essential oils, are not suitable for use in their raw state (due to volatilization, toxicity, poor solubility, degradation, etc.), different formulations may be used in order to increase the stability and efficiency of biopesticides [15]. Consequently, the review also includes an analysis of nano-sized formulations based on *Artemisia* spp.

## 2. *Artemisia* Compounds and Extracts with Pesticide Activity

### 2.1. Antifungal and Anti-Oomycete Activity

Pathogenic fungi produce almost 30% of crop diseases, threatening the health and food security of a growing human population dependent on substantial agricultural production [16]. Phytopathogenic fungi affect plants during their cultivation or after harvest, causing significant losses in crop plants. In addition, certain fungi (*Aspergillus* spp., *Fusarium* spp., *Alternaria* spp. etc.) produce mycotoxins that endanger the health of consumers through hepatotoxic, nephrotoxic, and carcinogenic effects or even cause death [15]. In an effort to find an ecological solution to this problem, numerous studies have assessed the antifungal effect of *Artemisia* species, focusing especially on volatile oil and compounds. Different methods of evaluation were used in vitro, in planta, or in field conditions, and the results were expressed in various ways: half maximal inhibitory concentration—IC_50_, minimal inhibitory concentration—MIC, minimum fungicidal concentration—MFC, median effective concentration—ED_50_, inhibition zone, and percent of inhibition (Table 1).

The in vitro antifungal activity was frequently determined by the agar diffusion test, which involves placing the tested plant extract in wells or paper discs on the agar plate previously inoculated with the pathogen [24,25]. Since essential oils diffuse less in the culture medium, it was preferred to include them in agar after prior solubilization, followed by inoculation of the pathogen [20,31,42]. Moreover, for volatile compounds, the fumigation method was used [20]. In vivo antifungal evaluations involved treating the plants with the tested compounds/extracts by spraying them followed by inoculation with the fungal pathogen or by including the compounds in the soil and then planting the inoculated seedling in the treated soil. The disease severity was assessed after a period of infection [21,50]. In situ antifungal efficacy against postharvest pathogens was determined by fumigation in the case of stored foods [39,40].

The extraction method influences the antifungal activity of the volatile oil, as can be seen from the investigation carried out by Julio et al. [18]: *A. absinthium* oil obtained by steam pressure extraction was more effective in inhibiting mycelium growth than that obtained by hydrodistillation, which was due to a different ratio of the major volatile compounds. Similarly, *A. argyi* essential oil obtained by simultaneous distillation–extraction had a higher antifungal activity compared to oils prepared by subcritical extraction or hydrodistillation. Although regardless of the extraction method, the oils had the same five major compounds, in the oil obtained by simultaneous distillation–extraction, the sesquiterpene compounds predominated [25]. Conversely, in the case of *A. chamaemelifolia* essential oil, the method of extraction—microwave-assisted hydrodistillation and classical hydrodistillation—had no influence on the inhibitory effect against the tested fungi. Both oils contained the same major compounds in comparable ratio [30].

The type of extract, the part of the plant used, and the time of harvest also influence the antifungal activity, as underlined in a study carried out with methanol, ethanol, and hexane extracts of *Artemisia annua* against *Aspergillus* niger and *A. flavus*. Whole plant extract was the most efficient in inhibiting the growth of the two fungi, regardless of the type of extract, compared to root, leaf, or stem extracts. Regarding the extraction solvent, ethanol extract had the highest inhibitory effect, followed by methanol and hexane, on both fungal species. Although the harvesting period of the plant had little influence on the antifungal activity, most of the extracts made with the plant collected during anthesis were more active [22].

From analyzing literature data, it appears that sesquiterpenes components of the oil have significant antifungal activity. Oxygenated sesquiterpenes were the major components of *A. khorasanica* volatile oil active against four soil-borne phytopathogenic fungi [37]. *Artemisia scoparia* essential oil, rich in sesquiterpenes, was more efficient in inhibiting mycelial growth and spore germination of *Alternaria solani* compared to *A. lavandulaefolia* and, especially, *A. annua* oils, where monoterpenes were the major compounds. Furthermore, the mode of volatile oil administration influences the outcome: *A. lavandulaefolia* oil was more effective when applied by fumigation than when mixed in the agar medium [20]. 

Alongside the sesquiterpenes, it seems that thujones present in high amounts in the volatile oil are associated with intense antifungal activity [32,42]. To prove this point, Shafi et al. [42] used a mixture of thujones (α-thujone, β-thujone, and fenchone) at the same concentration instead of *A. nilagirica* oil to achieve the same result against *Phytophthora capsici*—100% inhibition. Borneol was also tested in the aforementioned study and showed no antifungal activity. On the other hand, the antifungal property of *A. terrae-albae* essential oil against *Fusarium* spp. was associated with the presence of camphor, 1,8-cineole, camphene, α- and β-thujone, borneol, and the high content of oxygenated monoterpenes [48]. Other oxygenated monoterpenes, piperitone and carvone, were correlated with the antifungal activity on *Penicillium citrinum* and *Mucor rouxii*; the two ketones are major components of *A. herba-alba* volatile oil [33].

Some volatile compounds (L-camphor; DL-camphor, β-caryophyllene, and camphene) from *A. annua* oil were as efficient as synthetic antifungal products such as flutriafol and hymexazol against *Fusarium oxysporum* and *F. solani*, in vitro [21]. Different compounds isolated from the methanol extract of *A. incisa* were tested against *Aspergillus flavus* with various results: two monoterpenes and one phenolic acid derivative were more active compared to flavones and coumarins, the latter being less active [35].

Moreover, the synergistic action of essential oils and chemical fungicides was evaluated. Thus, *A. annua* essential oil combined with flutriafol exhibits additive inhibitory effect against *Fusarium solani*, while with hymexazol, it manifests synergistic activity on *F. solani* and additive action on *F. oxysporum* [21].

Most *Artemisia* extracts were tested on *Fusarium, Alternaria, Aspergillus,* and *Penicillium* species. Fungi have different susceptibility to varied antifungal compounds: for example, *Fusarium solani* was moderately sensitive to the action of isolated substances from *A. sieberi* (two sesquiterpene lactones and one methoxylated flavone), while *Alternaria alternata* and *Aspergillus niger* were resistant [45]. In an analogous manner, Aspergillus niger was sensitive to the methanol extract of *A. campestris* and resistant to *A. vulgaris* extract, despite similar quantities of flavonoids and phenolic compounds. Quercetin was reported in higher amounts in *A. campestris* extract and seems to be correlated with antifungal activity [28].

Few studies assessed the antifungal activity in vivo. Ma et al. [21] showed that the petroleum ether extract of *A. annua*, imitating the composition of the essential oil, decreased the incidence of infected *Panax notoginseng* plants when added in the culture mixture. *A. vulgaris* crude methanol extract exhibited weak to moderate antifungal activity against *Magnaporthe grisea, Thanatephorus cucumeris, Botrytis cinerea, Phytophthora infestans, Puccinia recondite,* and *Blumeria graminis* when tested on plants grown in greenhouse conditions [50].

Stored foods can be degraded by fungi such as *Alternaria* spp., *Penicillium* spp., and *Mucor* spp., which reduce their quality and make them unsuitable or even toxic for consumption. The use of chemical products for the control of postharvest pathogens endangers the environment, human health, and can induce resistance to fungicides. Such being the case, some investigations tried to estimate the reduction of postharvest fungal spoilage after treatment with *Artemisia* extracts. Fumigation of table grapes with *A. nilagirica* essential oil (200–300 µL) decreased the weight loss, berry shrinkage, and berry browning, increasing the shelf life for up to 10 days [39]. In addition, *A. nilagirica* volatile oil at a concentration of 1.4 μL/mL in airtight containers provided 71% protection from fungal contamination after 12 months of storage to millet grains [40].

In addition to the direct inhibition of postharvest phytopathogenic fungi, some studies also evaluated the mycotoxins suppression ability of plant extracts. For instance, *Artemisia herba-alba* keto-rich essential oil completely inhibited the toxin production (penicillic acid, terrestric acid, brevianamide A, aurantiamine, xanthomegnin) for *P. aurantiogriseum* at 0.44% and for *P. viridicatum* at 0.22% [32]. Similarly, *Artemisia nilagirica* essential oil inhibited the production of aflatoxin B_1_ by *Aspergillus flavus* toxigenic strain at 1 µL/mL. A common seed contaminant, aflatoxin B1 is a powerful human carcinogen and a serious health risk; it also contributes to food deterioration by lipid peroxidation [40]. In another experiment, *A. nilagirica* volatile oil (0.16 µL/mL) completely inhibited the production of aflatoxin B_1_ by *Aspergillus flavus* and ochratoxin A by *A. niger* and *A. ochraceus* [39].

The phytocompounds mechanism of action against fungi involves the inhibition of enzymes that control energy or structural compounds production, degeneration of fungal cell wall with loss of cytoplasm, and plasma membrane dysfunction. Due to their lipophilic nature, components of essential oils can penetrate cell walls, increase cellular membranes permeability and disturb the fungal cells metabolism, causing their death [11]. Monoterpenes delay sclerotic differentiation and promote the generation of lipid peroxides, which can lead to cell death, while phenols present in the essential oil bond to the active sites of fungal enzymes through their hydroxyl group [51]

In addition, spore germination and germ tube growth are negatively influenced by terpenes from the essential oil. *A. annua* volatile oil arrested mycelia growth and conidia germination of *Fusarium oxysporum* and *Fusarium solani* [21]. Electron microscope observations proved that *A. argyi* essential oil affected the cell morphology and the structure of cell walls in *Aspergillus niger* [25]. An earlier study showed that *Artemisia herba-alba* essential oil inhibited mycelium growth, spore germination, and sporulation of *Zygorrhynchus* spp., *Aspergillus niger* and *Penicillium italicum* [52].

The antifungal mode of action of *A. nilagirica* essential oil was investigated by Kumar et al. The fungal cells treated with 1.4 μL/mL volatile oil exhibited important deformity and shrinkage, detachment of plasma membrane from the cell wall, and development of lomasomes. At the same dose, *A. nilagirica* essential oil completely inhibited ergosterol synthesis in the cell membrane of *Aspergillus flavus* and provoked the leakage of Ca^2+^, K^+^, and Mg^2+^ ions from the cell [40].

It is worth mentioning that in addition to the secondary antifungal metabolites produced by plants, certain endophytic organisms present in *Artemisia* species are able to inhibit the development of phytopathogenic fungi. Thus, in the root, stem, and leaves of *A. argyi*, researchers identified endophytes (*Bacillus subtilis,* *B. cereus,* *Paenibacillus polymyxa*) that produce substances capable of inhibiting the growth of the mycelium of *Fusarium oxysporum,* *Magnaporthe grisea*, and *Alternaria alternata* [53].

### 2.2. Antibacterial Activity

Only a small number of studies investigated the effect of *Artemisia* spp. extracts on phytopathogenic bacteria. For instance, different *A. nilagirica* leaves extracts were tested in vitro against four phytopathogenic bacteria, *Erwinia* spp., *Clavibacter michiganense*, *Pseudomonas syringae,* and *Xanthomonas campestris*, which cause diseases in potato, tomato, leafy greens, carrot, onion, and green pepper. The hexane extract was the most efficient in inhibiting all tested bacteria with MIC of 32 µg/mL. The ethanol, methanol, diethyl ether, and chloroform extracts were moderately active against the four bacteria, while the petroleum ether extract was the least effective [54]. Methanol, ethanol, and chloroform extracts from leaves of *Artemisia parviflora* (1:6 *w*/*v*) were almost ineffective against *Xanthomonas vesicatoria* and *Ralstonia solanacearum*, with inhibition zones of 1 and 2 mm [55].

The essential oil of *Artemisia turanica* exhibited inhibitory activity at 2% (*v*/*v*) concentration against tumor galls induced by *Agrobacterium tumefaciens* on potato discs, but it did not demonstrate antibacterial activity in vitro against *A. tumefaciens* at the same dose [49]. In addition, the methanol extracts of roots, leaves, and flowers of *Artemisia fragrans* inhibited tumor growth in different percentages at 10, 100, and 1000 ppm. Leaves and flowers extract had the highest inhibition at all concentration (20, 38, 46%) compared to root extract (15, 24, 34%). No extract had any significant effect on the viability of *A. tumefaciens* when tested by agar diffusion assay [56].

Dadasoglu et al. [57] evaluated the antibacterial activities of essential oils, hexane, chloroform, acetone, and methanol extracts from the aerial parts of *A. santonicum*, *A. spicigera,* and *A. absinthium* against 25 plant pathogenic bacterial strains. *A. spicigera* essential oil was only active (MIC = 500 μL/mL) against *Erwinia amylovora*, *Pseudomonas syringae* pv. *syringae,* and *Xanthomonas axonopodis* pv. *vesicatoria*. The volatile oil of *A. absinthium* exhibited moderate activity (MIC = 250–500 μL/mL) against most of the phytopathogenic bacteria. *A. santonicum* essential oil was the most effective with MIC values 125–250 μL/mL on 22 out of 25 bacteria tested, with the exception of *Pseudomonas aeruginosa*, *P. cichorii,* and *Clavibacter michiganensis* subsp. *michiganensis*. None of the *Artemisia* solvent extracts manifested antibacterial activity on the tested strains. The main constituents of *A. absinthium* oil were chamazulene, nuciferol butanoate, nuciferol propionate, and caryophyllene oxide, while *A. santonicum* and *A. spicigera* oil shared similar major components: camphor, 1,8-cineole, cubenol, borneol, terpinen-4-ol, and α-terpineol. 

In the previously mentioned study, some constituents isolated from the essential oils were evaluated individually for their antibacterial activity. Caryophyllene oxide, camphor, borneol, and 1,8-cineole did not show activity against the phytopathogenic bacteria. Terpinen-4-ol inhibited the growth of all tested bacteria with MIC values ranging from 60 to 110 μL/mL and linalool blocked the development of 22 bacterial strains with MIC values in the 50–110 μL/mL domain. α-Terpineol was active (MIC = 60–70 μL/mL) only on *Pseudomonas cichorii*, *P. huttiensis*, *P. syringae* pv. *syringae,* and *Xanthomonas axonopodis* pv. *vesicatoria* [57]. 

The essential oil extracted from fresh leaves of *Artemisia proceriformis* manifested weak antimicrobial activity against four bacteria: *Erwinia carotovora* (MIC = 21.2 mg/mL), *Pseudomonas corrugate* (MIC = 21.2 mg/mL), *Pseudomonas syringae* (MIC = 5.31 mg/mL), and *Xanthomonas vesicatoria* (MIC > 42.5 mg/mL). The major component was α-thujone, in proportion of 66.9% [44].

Terpenes and phenolic compounds found in the essential oils are responsible for the intense antimicrobial activity. Terpenes have the ability to increase membrane permeability by infiltrating the phospholipidic bilayer; the damage to the bacterial membrane causes the loss of cytoplasmic components, which leads to cell death. Plant extracts are studied not only as inhibitors of bacterial growth, but also for the prevention of biofilm formation. Such is the case of *A. herba-alba*, *A. absinthium,* and *A. campestris* essential oils that can reduce biofilm formation by up to 70% [58].

### 2.3. Insecticidal Activity

Insects are the more diverse group of animals on Earth, and only 0.5% are considered pests. Nonetheless, herbivorous insects destroy every year one-fifth of the world’s crop production. Synthetic chemicals used to control insect pests are toxic to humans, animals, and the environment through accumulation. In addition, the development of insecticide resistance and the migration of harmful insects require the search for an alternative for plant protection. Considering these facts, botanical insecticides represent a viable substitute with low toxicity toward humans and the environment [59]. 

Plant-derived substances or plant extracts usually have a lower acute toxicity toward insects compared to synthetic insecticides. Nevertheless, their subacute toxicity was frequently noted and is important because it can limit insect spreading (diminished fertility, fecundity, vitality, or shorter lifespan) and decrease crop loss due to repellent, suppressant, or deterrent activity. These effects are generally called “antifeedant” and are manifested in insects by lower weight and body size, decreased fertility, and altered behavior [60].

*Artemisia* compounds can influence insects by direct contact or fumigation, can repel insects or keep them from feeding, or can hinder their reproduction. Volatile compounds can induce toxicity to insects via inhalation or direct contact by forming an impermeable film on the cuticle leading to suffocation. Some volatile components can penetrate through the cuticle, affecting cellular membrane function and oxidative phosphorylation [61]. Phytochemicals such as cinnamyl alcohol, eugenol, and trans-anethole can activate octopamine receptors, interfering with the normal activity of octopamine, a neurotransmitter, neuromodulator, and neurohormone in an invertebrate system [62]. Furthermore, volatile compounds can interfere with the γ-aminobutyric acid (GABA) receptor in insects [14]. Other studies reported the inhibition of acetylcholinesterase by 1,8-cineole, (-)-citronellal, limonene, α-pinene, pulegone, and 4-terpineol [63] or inhibition of adenosinetriphosphatase by essential oils [64]. In addition, plant substances may cause the suppression of cytochrome P450 in insects (the enzymes responsible for phase I metabolism of xenobiotics) and may alter various biochemical processes, which shift the balance of the endocrine system [14].

The activity of *Artemisia* compounds and extracts depends on the solvent used, the susceptibility of pest species to the active substance, the development stage of the insect, whether it is male or female, and the method of application. Table 2 lists the more recent studies on insecticidal activity of *Artemisia* genus. Essential oils and volatile compounds can be applied via fumigation, which is a procedure used frequently in the pest management of stored products. This method has obvious advantages such as the possibility to spread the substance evenly, even in unreachable places, and the ability to maintain an effective level of insecticides within a closed space [60]. Some of the shortcomings of natural insecticides are poor water solubility and rapid degradation in the environment, leading to low persistence and poor efficiency. To solve these problems, plant insecticides may be formulated as micro- and nanocapsules, nanoparticles, or nanoemulsions. These nanoformulations can increase the solubility, persistence, and stability of bioinsecticides, enhancing their activity and, at the same time, limiting their negative impact on the environment [65].

### 2.4. Nematicidal Activity

Plant parasitic nematodes cause severe yield losses in different crops, especially in tropics and subtropics. Frequent nematodes that affect plants include *Meloidogyne* (root-knot nematodes), *Pratylenchus* (lesion nematodes), *Xiphinema* (dagger nematodes), *Aphelenchoides* (foliar nematodes), *Globodera* (potato cyst nematodes), and *Heterodera* (soybean cyst nematodes). *Meloidogyne* species induce histological damages to roots, with the appearance of visible galls. Some phytoparasitic nematodes act as vectors for plant viruses, such as *Xiphinema* species [91].

Various *Artemisia* species were evaluated for nematicidal activity, some with promising results. For instance, *A. judaica* essential oil (1 μL/L) caused 85% mortality on *Meloidogyne javanica* second-stage juveniles and inhibited the hatching of eggs. The main component of the essential oil was artemisia ketone. In the same study, *A. arborescens* and *A. dracunculus* essential oils were poorly active on the root-knot nematode [92]. In vitro toxicity of *Artemisia annua* essential oil was evaluated against second-stage juveniles of *Meloidogyne incognita* and pre-adults of *Rotylenchulus reniformis* (reniform nematode). Concentrations of 500 and 250 ppm induced 100% mortality in both nematode species [93]. Moreover, there are reports of nematicidal activity exhibited by the alcoholic and aqueous extracts of *Artemisia annua* against *Meloidogyne incognita* and *Pratylenchus loosi* (tea root lesion nematode) [91].

*Artemisia herba-alba* essential oil produced 94.4% mortality on *Meloidogyne incognita* second-stage juveniles at 15 µg/mL and 100% mortality on *Xiphinema index* females at 2 µg/mL, after 24 h exposure. However, mixed-age infective specimens of *Pratylenchus vulnus* were more resistant to the activity of *A. herba-alba* essential oil with mortality values ranging from 56.8% to 67% after 24 to 96 h of exposure. The major components of the essential oil were cis- and trans-thujone, camphor, 1,8-cineole, trans-chrysantenyl acetate, and camphene. In an additional test, the three nematode species were exposed to various compounds of the essential oils of four plants, including *A. herba-alba*. Borneol and α-pinene manifested poor to moderate activity, while limonene lack activity on the three nematode species. Camphor exhibited a moderate nematicidal effect, whilst thymol and thujone (mixture of cis-thujone, 70% and trans-thuione) displayed strong activity against *M. incognita*, and less so on *P. vulnus* and *X. index*. The fact that the activity of the components of the volatile oil is weaker than that of the whole oil suggests a possible synergistic action of the mixture. In addition, soil treatments with 100 or 200 µg/kg *A. herba-alba* essential oil, by fumigation or application of water solution, significantly inhibited nematode density on tomato roots and in soil and also increased the plant biomass. Fumigation was proven to be more effective than drenching treatment [94].

*A. absinthium* essential oil (β-thujone 51% and linalyl acetate 24%) had over 99% mortality rate at 0.25 and 0.5% concentrations (**v*/*v**) against *Meloidogyne javanica* juveniles in an in vitro test. Furthermore, in vivo experiments were conducted in order to assess the ability of the essential oil to inhibit root-knot nematode development after being absorbed by the tomato plants. It was observed that spraying the oil on tomato leaves actually increased the number of galls and eggs in treated plants, and applying the essential oil into the soil at 0.25% and 0.5% concentrations did not lower the number of galls or nematode eggs in tomato plants. The authors believe that the nematicidal compounds could have been volatilized or degraded by microorganisms in the soil or by the plant, or possibly, the root exudates were modified by the absorbed essential oil, making the tomato plants more appealing to the nematodes [95]. In another study, commercially available *A. absinthium* volatile oil had only a slight effect on *Meloidogyne javanica* in vitro (the median lethal dose LC_50_ of 937 µg/mL at 48 h and 734 µg/mL at 72 h). The major components of the oil were borneol acetate, β-terpineol, 1,8-cineol, linalool, sabinene, and o-cymene [96].

The nematicidal activity of *Artemisia absinthium* hydrolate, a by-product of essential oil extraction, was evaluated on the root-knot nematode, *Meloidogyne javanica*. The hydrolate caused high mortality of second-stage juvenile and suppression of egg hatching, proving the ability of the *A. absinthium* hydrolate to penetrate the gelatinous matrix of eggs. In vivo tests showed a strong inhibition of juveniles’ penetration in the tomato roots. Soil treatment with *A. absinthium* hydrolate (60% and 20% concentrations) significantly reduced the reproductive capacity of root-knot nematode and the infection frequency. The main component of the hydrolate, responsible for the nematicidal activity, was identified as (5Z)-2,6-dimethylocta-5,7-dien-2,3-diol [97].

Kalaiselvi et al. [98] showed that essential oils of *A. nilagirica* plants collected from high and low altitude have different composition and different nematicidal activity against *Meloidogyne incognita* (LC_50/48h_ of 5.75 and 10.23 μg/mL, respectively). α-thujone, α-myrcene, and linalyl isovalerate were the main components of high-altitude *A.* *nilagirica* volatile oil, while the low-altitude plants produced an oil composed mostly of camphor, caryophyllene oxide, eucalyptol, humulene epoxide II, α-humulene, and β-caryophyllene. Experiments carried out in vivo by soil irrigation with the essential oil revealed that both volatile oils significantly reduced the infection of tomato plant (number of nematode juveniles and eggs) and enhanced plant growth (fresh weight of aerial parts and roots) at 20 µg/mL. Again, the effect was greater for the oil originated from high-altitude *A. nilagirica*. Moreover, the ethanol extract of flowering parts of *A. nilagirica* (1 mg/mL) exhibited nematicidal activity against *Meloidogyne incognita*, as reported by an earlier study [99].

Various hypotheses have been advanced as explanations for the nematicidal effects of essential oils: disruption of cell membrane permeability and obstruction of its functions, irreversible modifications of proteins structures from the nematode surface induced by aldehydes, inhibition of acetylcholinesterase with build-up of neurotransmitter in the central nervous system of the nematode followed by convulsion, paralysis, and death [11]. Research on *A. nilagirica* essential oil ascribe the nematicidal action to an increased generation of intracellular reactive oxygen species, activation of signaling pathway of apoptosis, and DNA damage prompting cell death [98].

In addition to the essential oils and their volatile compounds, few other substances from *Artemisia* genus have been tested for their activity against plant nematodes. Thirteen chemical compounds (apigenin, bonanzin, nepetin, dihydroluteolin, scopoletin, isoscopoletin, benzoic acid, β-sitosterol, γ-sitosterol, betulinic acid, friedelin, linoleic acid, and a long chain ketone) isolated from *Artemisia elegantissima* and *Artemisia incisa* were tested in vitro and in vivo for nematicidal activity against *M. incognita*. All phytochemicals significantly inhibited egg hatching and induced high mortality of second-stage juveniles at the tested concentrations (0.1, 0.2, and 0.3 mg/mL). Isoscopoletin was even more effective than the positive control carbofuran. In addition, application of the compounds as a root drench (0.1 mg/mL) on potted tomato plants caused a marked reduction of galls, galling index, and egg masses on plant roots, numbers of juveniles in the rhizosphere soil, and also improved tomato plant growth parameters (shoot and root length and weight). Isoscopoletin and apigenin were the most active compounds [100].

### 2.5. Herbicidal Activity

One of the most influential groups of plant secondary metabolites is the allelochemicals. They are released into the environment in order to affect the germination, growth, behavior, survival, and reproduction of competing plants, which is a process better known as allelopathy. They are produced mainly in the plant’s roots, seeds, flowers, and leaves, and their synthesis depends on the changes of the climate conditions as well as exposure to biotic or abiotic stress. Allelochemicals activity can be harmful or beneficial for the growth and survival of target species [101]. The destructive effect of allelochemicals is crucial for defending plants against herbivores and providing an advantage in the competition for resources [102]. In agroecosystems, allelopathy can influence weed management, and plant allelochemicals could be employed as bioherbicides in order to reduce the negative impact of chemical herbicides on the environment [103].

The allelopathic properties of *Artemisia* species are well known [104,105,106,107,108,109,110], so it was expected that numerous studies would investigate their herbicide potential on various weeds. Most researchers focused on the volatile oils, and only a few dealt with aqueous or alcoholic extracts (Table 3). The phytotoxic effect of essential oils is owed to multiple mechanisms of action: inhibition of cell division, decrease of mitochondrial respiration, reduction of photosynthetic pigments and photosynthesis, generation of radical oxygen species in excess and oxidative impairment, destruction of waxy cuticular layer, inhibition of enzymes activity, water uptake, and alteration of gibberellic acid content [102,111,112]. Most of these actions are correlated with the presence of oxygenated monoterpenes. For example,1,8-cineole and camphor inhibit DNA synthesis, cell proliferation, and elongation [113].

*Artemisia fragrans* essential oil inhibited seed germination and growth of *Convolvulus arvensis* at 1–4% concentration in a Petri dish and pot experiment. It significantly reduced the level of photosynthetic pigments (chlorophyll a, chlorophyll b, and carotenoids) and of antioxidant enzymes (catalase, peroxidase, ascorbate peroxidase, superoxide dismutase), as well as enhancing the production of hydrogen peroxide and malondialdehyde. It seems that volatile oil compounds—mostly oxygenated monoterpenes—inhibited the electron transport chain and affected the process of photosynthesis, leading to an increased production of oxygen reactive species. In turn, these intensified the lipid peroxidation of the cell membrane followed by electrolyte leakage [126]. 

Oxygenated monoterpenes were the major ingredients of *Artemisia sieversiana* essential oil (α-thujone 64.46% and eucalyptol 10.15%) that suppressed seedling growth of *Amaranthus retroflexus, Medicago sativa, Poa annua,* and *Pennisetum alopecuroides*. The experiment showed that the mixture of the major constituents, in the same ratio as found in the oil, was more phytotoxic compared to each individual compound, indicating a possible synergistic effect of α-thujone and eucalyptol [133].

Although oxygenated monoterpenes were the major constituents of *A. judaica* essential oils obtained by hydro-distillation and microwave-assisted extraction, the oil extracted by hydro-distillation exhibited greater phytotoxicity on *Lactuca sativa* seed germination and plant growth [36], showing that the extraction method impacts the phytotoxic activity of volatile oils.

Major constituents of *A. terrae-albae* essential oil were tested on seed germination, root and shoot growth of *Poa annua* and *Amaranthus retroflexus*. The phytotoxic effect of α-thujone, eucalyptol, camphor, and the mixture of these compounds was inferior to that of the essential oil, which suggests that probably other volatile components are causing the herbicidal activity of the oil [134]. α-Terpinen and β-pinene, compounds of *A. lavandulaefolia* essential oil, exhibited strong phytotoxic activity on seed germination test against eight target plants (Table 3), whereas β-caryophyllene and myrcene only inhibited *Achyranthes japonica* seed germination [128].

*Artemisia scoparia* essential oil inhibits germination and plant growth through the production of oxidative stress related to membrane disruption, increased lipid peroxidation, and buildup of hydrogen peroxide. It also interferes in cellular respiration and photosynthesis processes [132]. 

Field experiments in *Triticum aestivum* used pre-emergence application of *Artemisia vulgaris* aqueous extract (20% *w*/*v*) together with chlorsulfuron. This treatment permitted lowering the dose of the herbicide up to 80%, while manifesting an inhibitory effect of 70% against *Lolium multiflorum* [137]. Another field trial demonstrated that *A. argyi* water extract markedly suppressed the growth of weeds in *Chrysanthemum morifolium* field with no adverse effect on the growth of *C. morifolium*. The investigations showed that *A. argyi* inhibited weed growth and germination through inhibition of chlorophyll synthesis and photosynthesis [108]. Conversely, field treatment of *Triticum turgidum* L. subsp. *durum* Desf. with *A. absinthium* aqueous extract exerted a stimulating effect on weed presence and reduced wheat growth and yield [106]. 

The sensitivity of different weed species to a certain herbicide varies greatly. Among eight weeds tested in a study, *Amaranthus retroflexus, Echinochloa cruss-galli,* and *Reseda lutea* were more susceptible to the action of *A. vulgaris* essential oil, compared to *Rumex crispus, Agrostemma githago, Trifolium pretense, Chenopodium album,* and *Cardaria draba,* which were more resistant [135]. Similarly, *Parthenium hysterophorus* and *Ageratum conyzoides* were more vulnerable to the inhibitory effect of *Artemisia scoparia* volatile oil, in comparison with *Cassia occidentalis*, under laboratory conditions. In another test, *Echinochloa crus-galli* and *Parthenium hysterophorus* were more affected by post-emergence application of the oil [132].

The phytotoxicity of isolated compounds from *Artemisia annua* was evaluated against two monocots and five dicots (Table 3). The suppression of germination and seedling growth varies in the order: artemisinin>arteannuin B>artemisinic acid. *Raphanus sativus* was the most resistant to the action of tested compounds, followed by *Secale cereale*. The weaker activity of arteannuin B and artemisinic acid—molecules without an endoperoxide bridge—implies that the moiety is important for the phytotoxic effect [118]. Artemisinin reduces many physiological and biochemical processes in the target plant and affects mitosis by inhibiting microtubules formation [120,138].

The incorporation of artemisinin into soil inhibited the growth of above-ground lettuce plants, with EC_50_ = 2.5 mg/Kg sandy soil, but the germination was not arrested up to 100 mg/Kg soil [139]. Furthermore, adding *A. annua* leaves containing 0.81–0.22% artemisinin in soil led to the inhibition of *Zea mays* growth [140]. Artemisinin is phytotoxic in concentrations comparable to those of commercial herbicides and has a good activity in soil [110]. 

In vivo tests proved that artemisinin is a potent suppressor of photosynthetic activity through the formation of a highly reactive artemisinin-metabolite that is able to inhibit the photosynthetic electron flow [141]. Other investigations showed that artemisinin enhances the generation of radical oxygen species and lipid peroxidation, which leads to cell death and arrest of mitotic phases in *Lactuca sativa* seedlings [119]. When added to the culture medium of *Arabidopsis thaliana* seedlings, artemisinin (1, 2, 5, 20, 100 µM) reduced the root gravitropic responses, elongation of primary and lateral roots, root hairs density, and length. Furthermore, artemisinin diminished starch grain and auxin concentrations and affected auxin redistribution in root tips [142].

### 2.6. Activity on Non-Target Organisms

Since biopesticides and bioherbicides are of natural origin, they are considered to be less harmful to the environment and the health of applicators and consumers. Usually, plant-based formulations are mixtures of compounds, and they do not consist of a single substance, which should prevent resistance in target organisms. In addition, some phytochemicals are rapidly degraded in nature, so there is no risk of their accumulation in the environment, as is the case with chemical pesticides. Consequently, plant-based pesticides and herbicides are regarded as generally safe. Still, these products can affect the non-target organism directly or indirectly by influencing biodiversity and species interactions, so it is imperative to assess their safety [13,143].

Little information is available regarding the ecotoxicity of *Artemisia* compounds and extracts. Pino-Otin et al. [13] evaluated the toxicity of hydrolate and organic extracts from *A. absinthium* on three aquatic ecotoxicity indicator organisms: an invertebrate (*Daphnia magna*), a marine bacterium (*Vibrio fisheri*), and a unicellular freshwater alga (*Chlamydomonas reinhardtii*). The wormwood hydrolate, a by-product of essential oil extraction, is a promising biopesticide with nematicidal effect due to (5*Z*)-2,6-dimethylocta-5,7-dien-2,3-diol [97]. *A. absinthium* hydrolate caused acute toxicity on non-target organisms: *D. magna* (LC_50_ = 0.236%) > *V. fisheri* (LC_50_ = 1.85%) > *C. reinhardtii* (LC_50_ = 16.49%). Moreover, the wormwood ethanol extract was highly toxic to *D. magna* (LC_50_ = 0.093 mg/L). However, the effect of wormwood hydrolate on a river microbial community, composed mainly of Proteobacteria, was negligible, causing only small changes in metabolic diversity and a slight inhibition of bacterial growth. It is possible that natural freshwater microbial populations are more resistant to 2,6-dimethylocta-5,7-diene-2,3-diol action because of the modified bioavailability of compounds in the river water and particular sensitivity of the various microbial species [13].

The same *A. absinthium* hydrolate was tested on non-target soil organisms: natural microbial communities, the earthworm *Eisenia fetida*, and the plant *Allium cepa*. The hydrolate was toxic in low concentrations: it caused substantial inhibition of onion root growth (LC_50_ = 3.87% **v*/*v**), high mortality of the earthworm *E. fetida* (LC_50_ = 0.07 mL/g), and decreased bacterial metabolism (LC_50_ = 25.72% **v*/*v** after 1 day of exposure). All these effects were exhibited at inferior concentrations than those needed to contain the target organism. Probably, 2,6-dimethylocta-5,7-diene-2,3-diol is able to penetrate biological membranes and thus affect the survival and metabolic processes of soil organism from different trophic levels [13].

The methanol extracts of *Artemisia fragrans* manifested significant toxicity in the brine shrimp (Artemia salina) lethality assay, with ED_50_ = 19.7 ppm for the root extract and ED_50_ = 11.99 ppm for flowers and leaves extract [56]. In another study, the aqueous extracts from *Artemisia ordosica* leaves were tested on two algae from the biological soil crusts, *Chlorella vulgaris* and *Nostoc* spp. The less concentrated extract (1 g/L) stimulated *C. vulgaris* growth but did not significantly affect *Nostoc* spp., indicating that *C. vulgaris* might utilize the sugars and other carbon sources in the extract to promote self-growth. The highly concentrated extract (5 and 10 g/L) inhibited the growth of both algae [109].

The safety profile of the *Artemisia nilagirica* essential oil was determined in terms of mammalian toxicity on male mice (*Mus musculus*) and millet (*Eleusine coracana*) seeds viability. The essential oil showed low toxicity on mice (LD_50_ = 7528.10 µL/kg) and no effect on millet seed germination. Thus, the oil is suitable as a food preservative for both consumption and sowing purposes [40]. More so, *Artemisia nilagirica* essential oil did not cause any significant changes in the physicochemical and sensory properties of table grapes when applied by fumigation on the fruits [39].

*Artemisia absinthium* essential oil, a potential biopesticide, was evaluated for toxicity against non-target organisms: the honey bee (*Apis mellifera*) and tomato plant (*Solanum lycopersicum*). Honeybee toxicity (EC_50_ = 0.26 mg/cm^2^) is reached at lower concentrations of *A. absinthium* oil than the ones necessary for controlling the leaf miner *Tuta absoluta* (EC_50_ = 0.5 mg/cm^2^), but not at rates needed to control the whitefly *Trialeurodes vaporariorum* (EC_50_ = 0.08 mg/cm^2^). A similar phenomenon was noted for the phytotoxic effect on tomato; seed germination and root growth were inhibited at oil concentrations needed to control the leaf miner, but not the whitefly [66].

Investigations to date have shown that biopesticides derived from *Artemisia* are most likely to have some toxicity toward non-target organisms, and further studies are needed to assess the risk in natural communities in order to ensure the safe use of biopesticides in agricultural practices.

Choosing the right formulation can reduce toxicity as well as increase the stability and effectiveness of *Artemisia* biopesticides. For instance, terpenoids are lipophilic, volatile, and thermolabile compounds that are easily oxidized or hydrolyzed, so they can be affected during extraction, storage, and transport. Furthermore, after application onto plants, they volatilized quickly and start degrading, leading to short persistence and low efficacy in the field. These drawbacks can be overcome by a suitable formulation through encapsulation or nanoparticles synthesis. A product formulation is a homogeneous and stable mixture of components put together according to a specific procedure with the purpose of increasing the biological activity, stability, persistence, and efficiency, while decreasing the toxicity of the product. The selected formulation depends on the intended use and mode of application, the targeted phytopathogen, and the degradation factors present in the ecosystem [15].

## 3. Nanoformulations of *Artemisia*-Based Pesticides

### 3.1. General Notions on Nanostructures Used as Pesticides and Herbicides

Nanoscience and nanotechnology have great potential and numerous applications in many research areas, such as medicine, agriculture, electronics, catalysis, and water management [144,145]. 

Nanotechnology can be used to obtain nanoparticles, nanocapsules, nanoemulsions, nanogels, nanospheres, metal, and metal oxide nanoparticles that control or delay the delivery of active substances, adjust their absorption, and can prove to be more effective and environmentally safe and friendly. Nanoparticles (NPs) have specific sizes, a large surface area, different morphology, and high reactivity, which provide them with improved mechanical, electrical, optical, chemical, and magnetic properties, as well as with a different in vivo behavior. The production of nanocrystals is likely to increase the efficiency of pesticides at lower doses, followed by a decrease in soil and water pollution [146,147]. 

Given their chemical nature, nanomaterials can be classified into four major categories: carbon (comprising of carbon nanotubes, fullerenes, and graphene), ceramic (usually inorganic solids that consist of metal–oxide compounds), metal (Ag, Au, Cu, or Ni-containing nanomaterials), and polymeric compounds [145,148].

Nanotechnology presents various applications in agriculture through the production of nanofertilizers, nanoherbicides, nanofungicides, and nanosensors. Nanofertilizers ensure good development of the crop by promoting good absorption of micronutrients suitable for plant growing; they can be made of silica, titanium dioxide, zinc [149,150], copper [151], and even polymeric NPs [152]. Nanopesticides offer protection against biotic-type stresses; their main application is represented by encapsulation forms for the controlled release of pesticides, with improved selectivity and stability. Such compounds will cost less and have a longer duration of action [149]. 

Herbicides and insecticides are toxic substances with a long-term impact on the environment. Through nanoformulations, scientists intend to reduce their negative impacts and extend their life through controlled release, to provide a greater selectivity protecting other plant species, insects, and microorganisms, as well as to ensure their chemical protection to environmental factors such as degradation under UV radiation [153]. Different chemical compounds may be encapsulated in polymeric NPs in order to control the release rates of herbicides. Such controlled release is expected to work on competing weeds. For example, a nanoparticle system delivers a targeted herbicide molecule to a specific receptor in the roots of certain weeds, which enters through the roots and inhibits the glycolysis of nutrient reserves; the weed plant will no longer have access to food and will eventually die. The process is controlled by soil moisture and rainfall [154]. A study showed that the system comprising of paraquat (an extensively used nonselective herbicide) and alginate/chitosan NPs changes the release profile of the herbicide, the delivery being also influenced by soil interactions [153]. 

A nanoformulation made of chitosan/tripolyphosphate NPs was also used to encapsulate paraquat, and the system proved to be less toxic than the pure compound, which was efficient after encapsulation and showing good protection of other plant species [155]. Another system using silver modified with magnetite NPs and stabilized with carboxymethyl cellulose was studied. This system has shown an 88% degradation of the herbicide atrazine under controlled environment. Most NPs systems focus on the degradation of herbicides under different natural conditions [156,157]. Weeds are considered serious threats to global agricultural production as they compete with crops for nutrients, water, and light. Nanoherbicides prevent the regrowth of weeds in an eco-friendly manner. Different species of weeds exposed to SiO_2_NPs suffer alteration in germination, length, fresh and dry weights, pigments, and total protein content [158].

More than 90% of pesticides are either lost and accumulating in the environment or unable to reach target sites for the best pest control. Nanotechnology tries to design formulations with slow release of such substances. The main toxic effects of pesticides depend on the solubility, stability, decomposition under sunlight, and soil absorption [65,159]. Therefore, it is important to extend the study of such nanocompounds that can provide valuable nutrients and protection against pests (insects, bacteria, harmful plants) but can also induce stress in other species of the ecosystem or have negative effects on the antioxidant molecules profile of certain crops [160,161]. On the other hand, it is necessary to study and understand the exposure to plants and animals of nanoparticle-encapsulating pesticides in order to ensure a safe ecosystem–nanotechnology relationship [149].

Nanomaterials in pesticide formulations provide useful properties such as biodegradability, permeability, solubility, and thermal stability, which are indispensable to a sustainable agricultural environment [162,163]. Moreover, the controlled release of active ingredients reduces the total amount of used pesticides, thus protecting the environment and other plant species, as well as reducing costs. 

Different nanostructures were studied regarding their effects on plants and insects. Clay nanotubes used as carriers of pesticides showed extended release of substances, providing a better contact with minimum environmental effect [164]. Hydrophobic nanosilica is another example of such structures, which can be absorbed into the cuticle layer of insects upon contact, leading to their death [165]. Silica nanosphere formulations facilitate pesticides to enter the plant and reach the cell sap, exerting a systemic effect on insects such as aphids [166]. Moreover, these formulations alter the non-systemic behavior of pesticides and protect them from photodegradation [167]. 

Inorganic NPs such as ZnO, SiO_2_, TiO_2_, and AgNPs were studied for their plant protection potential [168]. For example, ZnONPs have been shown to provide effective growth control of fungi such as *Alternaria alternate, Aspergillus flavus, Fusarium graminearum*, *F. oxysporum, Penicillium expansum,* and *Rhizopus stolonifer* as well as of the *Pseudomonas aeruginosa* bacteria [168,169]. Moreover, TiO_2_ systems have been found to protect crops through a direct antimicrobial activity [170]. 

Different pesticide release systems were studied, and photocatalytic materials may find applications in the degradation of pesticides that are highly harmful for the environment [171]. A complete degradation by TiO_2_NPs was registered for many pesticides (e.g., chlorothalonil, chlorpyrifos, and cypermethrin) under UVA irradiation [172]. A distinct example is that of a Cu-doped ZnO system that showed high monocrotophos degradation [173]. 

Another class of such systems is represented by plant elicitors that are stress-inducing agents. They can be classified as biotic (fungal homogenates, insects, and microorganisms) and abiotic (temperature, light, salinity, wounds, metal ions) elicitors. It has been shown that several NPs can also act as elicitors, forcing the plant to defend itself by producing certain metabolites. A system based on cobalt NPs has shown a potential application for increasing artemisinin content in suspension cultures [174], while using AgNPs in combination with methyl jasmonate led to an improvement of the therapeutic qualities of *Calendula officinalis* L. [175].

Nanosystems using pesticides, fungicides, and herbicides represent an important step in reaching a sustainable agricultural development, having multiple potential applications in plant protection such as nanodiagnostics, disease management, pest and weed control, and pesticide remediation [170]. 

### 3.2. .Biosynthesis and Physicochemical Characterization of Metallic Nanoparticles (MeNPs) Using Artemisia spp. Extracts

Regarding the synthesis of NPs, two main approaches can be used: the top–down approach and the bottom–up approach, the main difference between them being the starting material [176,177]. Through the top–down approach, NPs are formed by reducing a bulk material into small units through chemical or physical methods, such as thermal milling, laser ablation [177], mechanical milling, sputtering and chemical etching [176]. Generally, these methods are quite easy to perform, but they involve high costs, high energy consumption, and can cause surface imperfections in NPs, thus altering their physicochemical properties [178,179,180].

On the other hand, the bottom–up approach starts from small units such as atoms and molecules that grow through self-assembly forming nuclei and, finally, NPs [176,177]. This category includes solid-state methods (physical and chemical vapor deposition), liquid state methods (sol–gel process, chemical reduction, hydro- and solvothermal method), gas state methods (spray and flame pyrolysis, laser ablation), biological methods, electrodeposition process, microwave and ultrasound techniques, supercritical fluid precipitation process, etc. [178]. Biological methods that can be found in this category use as starting materials plant extracts, fungi, bacteria, or yeasts, especially because they are environmentally and economically friendly, safe, biocompatible, and stable [176,178,181].

Using plant extracts to obtain MeNPs represents an approach that is gaining more and more attention, given the fact that plants have a widespread occurrence and are thus readily available [182]. Moreover, they contain important amounts of various metabolites functioning as both reducing and capping agents, which are responsible for the synthesis of homogeneous NPs, in significant amounts and in a short period of time [180,182,183]. Such NPs do not show pathogenicity, as in the case of fungi or bacteria [182].

#### 3.2.1. Biosynthesis of MeNPs Using *Artemisia* spp. Extracts

The synthesis of MeNPs using plants mainly involves collecting the plant, selecting an environmentally friendly solvent, obtaining the extract, and applying suitable reaction conditions for NPs synthesis, separating and finally purifying the formed NPs. The available literature provides several examples of MeNPs obtained using *Artemisia* spp. extracts, such as AgNPs, AuNPs, ZnONPs, CuNPs, and TiO_2_NPs. Therefore, some examples of conditions that can be used for the synthesis of AgNPs and AuNPs from different *Artemisia* species, such as *A. absinthium, A. abrotanum, A. afra, A. annua*, *A. arborescens, A. capillaris, A. haussknechtii, A. marschalliana, A. nilagirica, A. tournefortiana, A. tschernieviana,* and *A. vulgaris,* are presented in Table 4.

Generally, in the case of *Artemisia* spp., different parts of the plant such as leaves [184,185,186], stem barks [187], or aerial parts [188,189] collected from different sources are used in order to obtain the extract for MeNPs synthesis. The plant material is washed and dried, but it can also be used fresh [190] and afterwards ground, so as to use the obtained powder for extract preparation.

Extraction solvents such as water [191,192,193], ethanol [185], ethanol–water mixture [188,189,190], and even methanol [194] can be mixed with the plant material. The extract is obtained either by maceration [194], by heating or boiling for several minutes [184,191] or hours [187,195], or the mixture can be subjected to Soxhlet extraction [196]. However, it is recommended that high temperatures are avoided during heating, in order to prevent possible degradation of biomolecules that participate in the reduction process [197]. Most often, the mixture is further decanted or filtered through Whatman filter paper and stored in a cold place until further use.

**Table 4:** *Artemisia* spp. as sources of AgNPs and AuNPs.

*Artemisia* spp.	Plant Extract Conditions	MeNPs Type	MeNPs Synthesis Conditions	MeNPs Shape	MeNPs Size	Reference
*A. absinthium*	- extract: 1 g% - plant material: dried leaves powder - solvent: deionized water - extraction method: boiled, 5 min	AgNPs	- extract: metal salt ratio: 6:4 - metal salt: 2 mM AgNO_3_ - method: mixed - temperature: room temperature - time: 1 h	round	TEM: 5–20 nm	[192]
*A. absinthium*	- extract: 20 g% - plant material: dried leaves powder - solvent: distilled water - extraction method: boiled, 5 min	AuNPs	- extract:metal salt ratio: 1:5 - metal salt: 1 mM HAuCl_4_·3H_2_O - method: shaken by hand and left to react - temperature: 45 °C - time: 180 min	spherical, rectangular	SEM: <100 nm	[198]
*A. abrotanum*, *A. arborescens*	- extract: ≈6.6 g% - plant material: dried leaves manually minced - solvent: water:ethanol (1:1, *v*:*v*) - extraction method: 50 °C, 30 min	AgNPs	- diluted extract: metal salt ratio: 1:1 - metal salt: 1 mM AgNO_3_ - method: magnetic stirring - temperature: room temperature - time: 24 h	spherical	TEM: 20–30 nm DLS: 37 nm *A. abrotanum*; 30 nm *A. arborescens*	[197]
*A. afra*	- extract: 1.5 g% - plant material: dried leaves powder - solvent: distilled water - extraction method: heated, 1 h	AgNPs	- extract: metal salt ratio: 1:5 - metal salt: 1 mM AgNO_3_ - method: stirring - temperature: 90 °C - time: 1 h	spherical	TEM: ≈30.74 nm	[193]
*A. annua*	- extract: 5 g% - plant material: dried leaves powder - solvent: triple distilled water - extraction method: shaking, 2 h, 150 rpm, 60 °C	AgNPs	- extract:metal salt ratio: 1:9 - metal salt: 1 mM AgNO_3_ - method: shaken - temperature: room temperature - pH = 7 - time: 50–60 min	spherical	TEM: 20–90 nm DLS: 14.16 ± 8.56 nm	[199]
*A. annua*	- extract: 2 g% - plant material: dried leaves powder - solvent: deionized water - extraction method: boiled, 5 min	AgNPs	- extract:metal salt ratio: 1:10 - metal salt: 5 mM AgNO_3_ - method: stirred - temperature: room temperature - time: 10 min	spherical	TEM: 30–50 nm	[191]
*A. annua*	- extract: 2 g% - plant material: dried leaves powder - solvent: deionized water - extraction method: boiled, 5 min	AuNPs	- extract:metal salt ratio: 1:10 - metal salt: 5 mM HAuCl_4_·3H_2_O - method: stirred - temperature: room temperature - time: 10 min	spherical, triangular	TEM: 15–40 nm	[191]
*A. annua*	- extract: 10 g% - plant material: fresh chopped leaves - solvent: 50% ethanol - extraction method: 60 °C, 10 min	AgNPs	- diluted extract:metal salt ratio: 1:9 - metal salt: 2 mM AgNO_3_ - method: mixing - temperature: room temperature - time: 5 min	spherical	TEM: 7–27 nm	[190]
*A. capillaris*	- plant material: dried aerial parts powder - solvent: water	AgNPs	- metal salt: 1 mM AgNO_3_ - method: oven incubation - temperature: 80 °C - time: 4 h	spherical, triangular, hexagonal, spheroidal, amorphous	AFM: 29.71 nm	[200]
*A. capillaris*	- extract: 10 g% - plant material: dried aerial parts powder - solvent: deionized water - extraction method: sonication, 3 h	AuNPs	- metal salt: 0.25 mM HAuCl_4_·3H_2_O (concentration in final solution) - method: incubation in a dry oven - temperature: 80 °C - time: 1 h	spherical, triangle, rods	TEM: 16.88 ± 5.47–29.93 ± 9.80 nm DLS: 26.9–41.3 nm	[201]
*A. haussknechtii*	- extract: 8 g% (fresh and dried) - plant material: dried leaves powder - solvent: double distilled water - extraction method: boiled at 90 °C, 30 min	AgNPs	- extract:metal salt solution: 1:9 - metal salt: 0.1 M AgNO_3_ - method: stirred - temperature: room temperature - time: 24 h	triangle	XRD: 47 nm SEM: 10.69 ± 5.55	[202]
*A. marschalliana*	- extract: 10 g% - plant material: dried leaves powder - solvent: deionized water:ethanol (1:1, *v*:*v*) - extraction method: boiled, 20 min	AgNPs	- extract:metal salt ratio: 1:25 - metal salt: 0.01 mM AgNO_3_ - method: stirred - temperature: room temperature - time: 5 min	spherical	TEM: 5–20 nm FE-SEM: 5–50 nm	[203]
*A. nilagirica*	- extract: 10 g% - plant material: fresh leaves cut into very fine pieces - solvent: distilled water - extraction method: boiled at 60 °C, 60 min	AgNPs	- extract:metal salt solution: 1:9 - metal salt: 1 mM AgNO_3_ (concentration in final solution) - method: held in the dark - temperature: room temperature - time: 60 min	spherical to irregular shape	XRD: 6.723 nm SEM: ≤30 nm	[204]
*A.* *tournefortiana*	- extract: 10 g% - plant material: dried aerial parts powder - solvent: water:ethanol (1:1, *v*:*v*) - extraction method: boiled, 30 min	AgNPs	- extract: metal salt ratio: 1:25 - metal salt: 0.001 M AgNO_3_ (concentration in final solution) - method: stirring - temperature: room temperature - time: 10 min	spherical	SEM: 22.89 ± 14.82 nm	[189]
*A.* *tschernieviana*	- extract: 10 g% - plant material: dried aerial parts powder - solvent: water:ethanol (1:1, *v*:*v*) - extraction method: boiled, 30 min	AgNPs	- extract: metal salt ratio: 1:1 - metal salt: 0.01 mM AgNO_3_ - method: stirring - temperature: 30 °C - time: 5 min	spherical	SEM: 5–50 nm	[205]
*A. vulgaris*	- extract: 1 g% - plant material: dried leaves powder - solvent: methanol - extraction method: macerated 3 times, room temperature	AgNPs	- extract: metal salt ratio: 1:1 - metal salt: 20, 50, 100 mM AgNO_3_ - method: magnetic stirring - temperature: room temperature - time: 15 min agitated and 2 h (incubation)	spherical	TEM: 25 nm SEM: 27–53 nm	[194]
*A. vulgaris*	- extract: 10 g% - plant material: dried leaves powder - solvent: distilled water - extraction method: boiled at 60 °C, 30 min	AuNPs	- extract:metal salt ratio: 1:9 - metal salt: 1 mM HAuCl_4_·3H_2_O - method: mixed and left to react - temperature: room temperature - time: 20 min	spherical, triangular, hexagonal	TEM: 50–100 nm DLS: 89.76 nm XRD: 6.1 nm	[206]

The synthesis of MeNPs is achieved by mixing a metal salt of different concentrations with the plant extract, in different proportions, for a certain period of time at different temperatures. In order to separate MeNPs, the suspension is centrifuged (e.g., 4000 rpm [193], 13,000 rpm [189,205]) for different periods of time (e.g., 20 min [203], 1 h [193]), followed by repeated washing so as to remove unreacted metal ions and small biomolecules [207], and then, it is dried in an oven at low temperature.

The reaction rate influences the phytofabrication, which along with the shape, size, and distribution of MeNPs depends on factors such as temperature, pH, salt and extract concentrations, and reaction time. The stability of MeNPs also depends on the temperature and reaction time [199,208]

Taking into consideration that most papers that use *Artemisia* spp. to obtain MeNPs and study the production of AgNPs, the present review will focus more on this type of nanoparticles.

The pH is a crucial factor for the synthesis of AgNPs, its variation leading to the modification of charged biomolecules, thus influencing their ability to reduce Ag ions [199,207]. For example, in the case of AgNPs obtained using *A. annua*, the pH was investigated in the 3.0–9.0 range. The results showed that at pH 3.0 and 5.0, there is no reduction of Ag ions, while at neutral (7.0) and alkaline pH (9.0), respectively, small AgNPs are obtained [199]. Therefore, it was confirmed that the pH is also responsible for variations in the size and morphology of NPs [207,209]. 

In order to analyze the influence of temperature on nucleation and AgNPs size, Anush et al. compared the UV-Vis spectra of the reaction mixture at room temperature, at 40 °C and at 60 °C, respectively. The intensity of absorption peaks showed that AgNPs synthesis is achieved in a shorter time at higher temperatures, and the obtained peak is sharper, while at room temperature, the synthesis proceeds more slowly, and the absorption peak is broader [199]. The sharpness of the surface plasmonic resonance (SPR) band can be attributed to smaller AgNPs [209], while a broad peak suggests that AgNPs are polydisperse [192]. Polydispersity can be explained taking into account the variety of biomolecules present in the extract, which have different reducing capacities and, thus, influence the nucleation and growth of AgNPs [192].

Other factors that must be taken into account are the extract and silver salt concentrations. During the synthesis of AgNPs using *A. annua*, silver nitrate (AgNO_3_) solutions in the 0.5–4 mM range and 2.5–15 g% extract concentrations were tested. The maximum amount of AgNPs was obtained at a concentration of 2 mM AgNO_3_. Regarding the influence of the extract concentration, the synthesis of AgNPs increased until a 5 g% concentration, followed eventually by a decrease [190].

The synthesis of AgNPs using an *A. afra* extract is a good example for highlighting the influence of the reaction time. In this case, it was observed that with the increase of the reaction time, the intensity of the absorption peak corresponding to SPR increased, thus demonstrating a rise in the synthesis rate. The AgNPs synthesis was complete after 30 min, as demonstrated by the overlapping peaks of the UV-Vis spectrum at 45 and 60 min. A continuation of the reaction after 60 min demonstrated a shift of the peak to shorter wavelengths, which could be explained by a slight reduction in the AgNPs size [193]. This is because, generally, the absorption peaks at shorter wavelengths point to smaller particle sizes, while absorption peaks at longer wavelengths indicate an increase in particle size [192].

Other confirmations of the influence of the factors discussed above can be seen in Table 4. For the same species of *Artemisia*, different extract concentrations and extraction conditions, as well as different AgNO_3_ concentrations and extract:AgNO_3_ ratios, led to the obtaining of AgNPs in a 5–60 min range.

Furthermore, the scientific literature contains data on even higher reaction rates, which led to the obtaining of AgNPs in as much as 2 min when using *A. quttensis* [188], but also, on lower reaction rates that can take up to 24 h for the reaction to be completed, especially in the case of AgNPs obtained using *A. abrotanum* and *A. arborescens* [197].

In the case of AuNPs, the reaction conditions influence the quantity, shape, and size of the obtained nanoparticles. An example is that of AuNPs synthesized using *A. dracunculus*. The extract was obtained by a microwave digestion system at 80 °C for 220 s, which was followed by cooling down for 400 s. The synthesis of AuNPs was performed in a reactor by continuously heating and stirring at 80 °C. The studied conditions were represented by an extract concentration of 1–5% (*v*/*v*), a 0.05–5 mM gold salt concentration in a pH range of 2.8–5, and a reaction time of up to 60 minutes. It was proved that 1–2% extract concentrations were ineffective, while for concentrations between 3 and 5%, the characteristic SPR peak is displaced from 850 to 700 nm (which reflects an increase in nanoprism edge length) with the increase of extract concentration and decrease in the size of triangular nanoparticles. The 0.05 mM and 0.275 mM concentrations of chloroauric acid (HAuCl_4_) proved to be suitable for obtaining spherical nanoparticles, while at higher concentrations, the shape varied between spherical, hexagonal, and triangular. For concentrations higher than 1 mM HAuCl_4_, the size of all nanoparticles increased. At a pH lower than 4, the formed NPs are mostly triangles, while at a pH higher than 5, no triangular AuNPs were formed [210]. It can also be speculated that at low pH values, there is a tendency of aggregation rather than of nucleation [209].

The established optimal reaction time was between 10 min for AuNPs obtained using *A. annua* [191] and 20 min for AuNPs obtained using *A. vulgaris* [206].

Lately, zinc nanoparticles (ZnONPs) have also been gaining more and more attention due to a wide range of applications and, as well as for other MeNPs, green synthesis provides valuable results in this case as well.

Various conditions for the synthesis of ZnONPs nanoparticles using *Artemisia* spp. have been identified. One can start from a classic aqueous extract obtained by heating and stirring at 80 °C for 20 min and at 46 °C for 24 h, as in the case of *A. annua* stem barks [187] or by distillation at 110 °C of a jelly paste obtained from plant leaves and stems ground with distilled water, as in the case of *A. pallens* [211]. Moreover, a methanolic extract obtained by shaking incubation at 25 °C for 48 h was also used in the case of *A. aucheri* aerial parts [195].

A zinc salt (zinc nitrate, zinc acetate) was added to the extract either in solid state [195] or in solution [187,196,211], followed by stirring for minutes [195] or hours [187,211], at room temperature [211] or at higher temperatures [187]. The obtained precipitate was subjected to heating at high temperatures in order to attain purity. 

#### 3.2.2. Physicochemical Characterization of MeNPs Obtained Using *Artemisia* spp. Extracts

The first indication of the conversion of Ag^+^ to Ag^0^ is represented by the visual change of the mixture color from clear [184] or yellow [191,193,194] to yellowish brown [184], reddish brown [189,193,205], light brown [190], dark brown [188,191,199,203], or black brown [194]. In the case of AuNPs, the reduction of Au^+3^ to Au^0^ is demonstrated by the change in the color of the solution from yellow to purple-red (violet) [206], pinkish violet [210], or dark pink [191]. For CuNPs and TiO_2_NPs prepared from *A. haussknechtii*, the reduction of CuSO_4_ and TiO(OH)_2_ is observed through the change of the color to pale green (CuNPs) or milky (TiO_2_NPs). The color modifications are determined by the SPR band of MeNPs, which is caused by the free electrons on the surface of the NPs and their combined vibration in resonance with the light wave [186].

The AgNPs synthesis and stability are usually monitored by recording the UV-Vis spectrum during the reaction, detecting the characteristic SPR peak in the 400–450 nm range: 410 nm [199], 420 nm [189,194], 430 nm [185,203], or 450 nm [186]. For other types of MeNPs, the appearance of the SPR band at more than 500 nm demonstrates the synthesis of AuNPs [191,206] with an increase of the absorbance intensity in time [191]. In other cases, the broad absorption peak at 330 nm demonstrates the monodisperse nature of the formed ZnONPs [187], and the broad peak found between 200 and 300 nm in the case of CuNPs reflects a wide size distribution, while the intrinsic band gap absorption at a wavelength smaller than 400 nm is attributed to TiO_2_NPs formation [202]. 

In order to demonstrate that biomolecules are involved in the reduction of metal ions, FTIR analysis is used, which allows the comparison of the FTIR spectrum of the extract with that of the synthesized MeNPs. Taking into account the wavenumber values of the FTIR spectra of *Artemisia* spp. extracts, the following absorption bands can be generally identified: O-H stretching vibrations attributed to phenols and alcohols [188,203,205], C-H stretching vibrations attributed to alkanes [185,188,205] or benzene rings [185], C = O stretching vibrations for amide carbonyl groups found in proteins [185,197] and enzymes [197], C-O stretching vibrations [188,193], CH_2_ bending vibrations [185,193], glycosidic or ether C-O-C bonds and C-N stretching vibrations for aromatic amines [203,205], stretching vibrations of the C-H bond adjacent to a quinone moiety, stretching vibrations of the C = C bonds that are adjacent to a quinone system or found in an aromatic system [197].

The presence of the same bands in the FTIR spectra can demonstrate that the respective biomolecules are present on the surface of NPs, while the shifts to smaller or larger wavenumbers demonstrate the interaction of the components with Ag atoms [193]. Khalil et al. identified a new band in the FTIR spectrum of the AgNPs compared to the initial *A. tschernieviana* extract spectrum at 2362 cm^−1^, which was probably due to a new alkane C-H stretching vibration [205]. Moreover, Mousavi et al. identified a band at 1382 cm^−1^ attributed to a stretching vibration of the N = O bond found in the nitro group, which is formed by the oxidation of the amino group and the reduction of Ag ions [185] in the case of AgNPs obtained using *A. turcomanica*. 

The comparative FTIR analysis of the spectra of extracts and AuNPs reveals the same functional groups (O-H, C = O, and C-O) [206,210], that were attributed to some phenolic acids and flavonoids present in *Artemisia* extracts (chlorogenic acid, caffeic acid, rutin, tannic acid, salicylic acid, ascorbic acid, 2,5-dihydroxybenzoic acid, ethyl p-anisate, niacin), but also N-H bonds [210]. Therefore, such compounds are responsible for the reduction process and can be adsorbed/complexed on the surface of AuNPs [201].

In addition, the FTIR spectrum of ZnONPs shows a peak at 550 cm^−1^ [195] or 478 cm^−1^ that can be attributed to the Zn–O bond [196,211]. Another example demonstrates that during the FTIR analysis of CuNPs and TiO_2_NPs, in addition to the shifts observed in the MeNPs spectra, some prominent peaks appear compared to leaf extract [202].

Therefore, biomolecules containing carbonyl and hydroxyl groups as well as carboxyl and amide bonds have a greater capacity to participate as reducing agents in the MeNPs synthesis [189,193,203]. The explanation given by Elemike et al. would be that metal ions can form an intermediate complex with free radicals present in biomolecules, which subsequently undergo an oxidation process to keto forms with the consequent reduction of metal ions to MeNPs [193].

The synthesis of MeNPs using *Artemisia* spp. is due to biomolecules present in these plants, such as flavonoids, terpenoids, coumarins, sterols, enzymes, polyphenols, alkaloids, carbohydrates, and amino acids [187,188,189,206]. Khalili et al. demonstrated that compounds such as cedreanol, 6,10-dodecatrien-3-ol,3,7,11-trimethyl, α-bisabolol, phytol, and spathulenol can participate in the synthesis of AgNPs from *A. tschernieviana* [205].

Consequently, antioxidant metabolites and plant enzymes that have the role of preventing oxidation and cell damage can act as reducing agents and thus be used as scaffolds to direct the MeNPs synthesis. Among these metabolites, flavonoids have an important reducing potential on metal ions mainly through their ability to donate electrons or hydrogen atoms and change the keto group to enol [208]. For phenolic acids, the reducing capacity depends on their structure and can be attributed to nucleophilic aromatic rings, a phenolic hydroxyl group, which as a result of interaction with metal ions undergoes an oxidation process in the case of gallic acid or electron delocalization between the aromatic ring and the propanoic chain for caffeic acid. Proteins can also participate in the synthesis of MeNPs through carbonyl, hydroxyl, and/or amino groups [207].

Another aspect confirmed by FTIR analysis is that biomolecules from plant extracts can form a layer covering the MeNPs [197], which prevents the agglomeration of nanoparticles and hence contributes to their stability in the environment. Therefore, biomolecules present in extracts act as both reducing and stabilizing agents for the synthesized MeNPs [207].

In order to confirm the stability in aqueous medium, the surface electric charges measured through the zeta potential are usually determined for MeNPs obtained using *Artemisia* spp. [197]. In this regard, some of the obtained values are −5 mV for AgNPs obtained using *A. tschernieviana* [205], −20.6 ± 0.89 mV for AgNPs obtained using *A. quttensis* [188], −31 mV for AgNPs obtained using *A. marschalliana* [203], −30 mV for AuNPs obtained using *A. dracunculus* [210], −19.3 mV for AuNPs obtained using *A. vulgaris* [206], and −38 mV for ZnONPs obtained using *A. aucheri* [195]. Negative zeta potential values indicate a strongly negative surface charge and implicitly no significant tendency of aggregation [195]. The negative values of the surface charge potential can be explained by the presence of biomolecules in the extract that act as capping agents [205], in which a greater negative surface charge value suggests a higher stability [203].

X-ray diffraction (XRD) is a technique that can be used for structural analysis of MeNPs. Generally, for AgNPs, in the 10°–80° 2*θ* range, four diffraction peaks are observed around 38°, 44°, 64°, and 77°, which correspond to the (111), (200), (220), and (311) planes of the face-centered cubic silver crystal, demonstrating the nanocrystalline nature of AgNPs [185,188]. For AgNPs obtained using *A. annua*, Khatoon et al. recorded a 5th diffraction peak around 81°, which was indexed to (222) orientation. The XRD analysis of the AuNPs revealed approximately the same values for diffraction peaks as in the case of AgNPs that correspond to (111), (200), (220), and (311) planes, confirming the crystalline nature of AuNPs. The XRD pattern of ZnONPs showed seven different peaks, with a 4–9 nm crystallite size, which demonstrates the material’s nanostructure [195]. For CuNPs, the XRD analysis highlighted 11 diffraction peaks around 33°, 36°, 39°, 48°, 54°, 58°, 63°, 67°, 69°, 74°, and 77°, corresponding to (110), (002), (111), (202), (020), (202), (113), (311), (113), (311), and (004) planes, while for TiO_2_NPs, there are 12 diffraction peaks around 25°, 37°, 48°, 54°, 56°, 58°, 63°, 69°, 70°, 75°, 77°, and 84°, corresponding to (101), (004), (200), (105), (211), (204), (116), (220), (215), and (303) planes, demonstrating the crystal structure of MeNPs [202]. The unassigned peaks observed in some cases are probably due to proteins present in the extract that crystallize or to bioorganic matter found on the surface of nanoparticles [191].

Another important aspect is related to the shape, size, and morphological structure of MeNPs, which influence their functionality and toxicity on the environment and human body [199]. Transmission electron microscopy (TEM), scanning electron microscopy (SEM), and dynamic light scattering (DLS) techniques are used for such determinations. As seen in Table 1, AgNPs obtained from *Artemisia* spp. have a spherical shape and a wide particle size distribution. Unlike AgNPs, which are mostly spherical, the morphology for AuNPs obtained using *A. dracunculus* and *A. vulgaris* determined by TEM is diverse: spherical, triangular, and hexagonal [206,210], while in the case of AuNPs synthesized from *A. annua*, almost all are spherical with a few triangular or irregularly shaped particles [191]. As previously mentioned, the particle size is strongly influenced by the reaction conditions. Basavegowda et al. proved that spherical particles are smaller than triangular ones and can agglomerate to form slightly larger non-spherical particles [191]. On the other hand, the size distribution and morphology of ZnONPs showed spherical or granular [187,195] NPs with an average size of 20–30 nm or hexagonal-shaped NPs with an average size of 50–100 nm [211]. For these examples, a tendency of agglomeration or clustering was observed [187,195]. SEM analysis indicates a spherical shape for CuNPs and TiO_2_NPs, but the average sizes differ: 35.36 ± 44.4 nm for CuNPs and 92.58 ± 56.98 nm for TiO_2_NPs [202]. 

For the determination of the elemental composition of MeNPs, the spectrum obtained by energy-dispersive X-ray spectroscopy (EDX) is generally used. The presence of a typical intense signal at ≈3 keV, due to SPR, confirms the existence of metallic silver in the case of AgNPs [185,189,191,202]. Other peaks representing different valence states of Ag may appear near the intense optical absorption peak [194]. For other types of MeNPs, strong signals can be identified in the EDX spectrum, thus confirming the existence of metallic gold [191,206], the presence of mainly Zn and O [195], or the existence of copper (0.96 keV) and titanium (4.56 keV) atoms [206]. For AuNPs obtained using *A. vulgaris*, the formation of bimetallic cluster can be observed rather than phase-separated monometallic nanoparticles [206]. Other signals that appear on the spectrum, such as that of chlorine, represent another confirmation of the presence of organic moieties with capping role in the extract [188,203].

### 3.3. Applications of MeNPs Obtained Using Artemisia spp. as Nanopesticides and Nanoherbicides

The synthesis of MeNPs using *Artemisia* spp. is becoming an important source of potential applications in many fields. In addition to widely studied properties such as antibacterial [184,188,189,191,202,203], antioxidant [186,188,193,203] and anti-cancer [188,189,203,205], the use as nanopesticides and nanoherbicides is also being investigated, with few results being reported so far, even if *Artemisia* spp. are recognized to have such biological activities as well [104,212,213,214].

One of the most pathogenic species of nematodes for most crops is *Meloidogyne* spp. *A. judaica* has been shown to have antifeedant (against *Spodoptera littoralis*) and fungicidal properties (on several pathogenic fungi), as well as the ability to determine the immobilization of the 2nd juvenile stage of *Meloidogyne javanica* through its essential oil components (especially piperitone and trans-ethyl cinnamate) [215,216].

To increase nematicidal efficacy, Soliman et al. prepared and compared AgNPs obtained using *A. judaica* extracts in different solvents (petroleum ether, ethyl acetate, ethanol) with AgNPs obtained using the essential oil and AgNPs prepared using a reference pesticide and compared the extracts. LC_50_ values showed that all types of nanoparticles were more toxic to the second juvenile stage of *Meloidogyne incognita* than the extracts. Comparing the activity of AgNPs obtained using the reference pesticide and those obtained using the extracts or the essential oil, respectively, proved that the NPs obtained using the reference pesticide had the highest inhibitory effect. As for AgNPs obtained from *A. judaica*, their activity increased up to 3-fold, being influenced by the extraction solvent (AgNPs obtained using petroleum ether extract> AgNPs obtained using ethyl acetate extract> AgNPs obtained using essential oil> AgNPs obtained using ethanol extract). Regarding the inhibition of egg hatchability by extracts and AgNPs, the results were similar, with NPs having a better activity compared to extracts, and among the NPs, the best results being obtained when using petroleum ether as solvent. The explanation could lay in the chemical composition of *A. judaica* extracts and of AgNPs. The chemical analysis revealed that the major components of the petroleum ether extract were 6-octadecanoic acid, n-hexadecanoic acid, 1,3-dimethylbenzene (m-Xylene), bis (2-ethylhexyl)phthalate, octacosane, 9,11-dimethyl-6H-indolo-quinline, nonacosane, cyclohexanol,3-ethenyl-3-methyl-2(1-methylethenyl)-6(1-methylethyl), and cyclohexanol-3-ethenyl-3-methyl-2-(1-methylethenyl)-6-(1-methylethyl, while for the corresponding NPs, the major compounds were 4-trimethyl-yciclo-hept-’-en-3′-yl]-3-buten-2-one, berkheyaradulene, β-caryophyllene, and allo-aromadendrene, which were found to be 20 to 30-fold increased [217].

Another example is that of AgNPs synthesized from *A. absinthium*, which have been tested against some oomycetes of the *Phytophthora* genus *(P. capsici, P. cinnamomi, P. infestans, P. katsurae, P. palmivora, P. parasitica,* and *P. tropicalis*), which are responsible for many crop diseases and are known for developing resistance to fungicides. The studied AgNPs have been shown to have high potency and efficiency on mycelial growth, spore germination, germ tube elongation, zoospore production, and spore encystment, especially for *P. parasitica* and *P. capsici.* Furthermore, in the case of treating tobacco plants with AgNPs it was observed that not only did they prevent infection and improve plant survival, but they also had no adverse effects on plant growth or anatomy [218].

Due to the contained phytochemicals, *Artemisia* spp. could also be used for the mosquito larvicidal activity. There are several studies investigating the effectiveness of MeNPs against different developmental stages of *Anopheles stephensi* and *Aedes aegypti*, with the possibility of malaria and dengue fever prevention. Nalini et al. conducted research on the activity of AgNPs synthesized from *A. nilagirica* on larvae and pupae of the two vector species compared to that of the aqueous extract. The results showed that AgNPs have better larvicidal and pupicidal properties compared to the extract. For both species, the higher rate of susceptibility was observed in pupa with a linear increase from the 4th to 1st stage (except for the extract against *Aedes aegypti*, where the 2nd stage required a higher dose than the 1st stage in order to cause lethal effect). Another research study tested the insecticidal action of AgNPs obtained using an *A. herba-alba* extract on *Anopheles stephensi, Aedes aegypti*, but also against *Culex pipiens* and *Culex quinquefasciatus* [219]. Hydroxycinnamic derivatives, flavonoids, and saponins were identified in the extract, which can influence the toxicity. In this case, the AgNPs showed an important larvicidal and adulticidal activity against the tested strains [212]. 

The exact mechanism of the AgNPs larvicidal effect is still unknown and is currently being researched. Larval mortality may appear either because of penetration of AgNPs through treated larval membranes and interaction with cell membranes, because of cell death resulting from the inactivation of enzymes and peroxide generation when AgNPs reach the midgut epithelial membrane, or because of the interaction of AgNPs with sulfur and phosphorus found in cell membranes [204]. The explanation for the accumulation of AuNPs in the midgut region of the larvae, which is not observed in the case of exposure to essential oil, and the implicit possible stronger larvicidal action is given by Sundarajan and Kumar [206]. Their study on the larvicidal activity of AuNPs synthesized from *A. vulgaris*, compared to that of the essential oil against 3rd and 4th instar larvae *Aedes aegypti*, confirmed the better activity of AuNPs regarding damage to the midgut, epithelial cell, and cortex, after 24 hours of exposure [206,220]. It is considered that β-caryophyllene may conjugate with Au ions and thus present larvicidal action [206].

### 3.4. Other Types of Nanosystems Based on Artemisia spp. Used as Pesticides and Herbicides

A novel alternative approach used for larvicidal activity, besides MeNPs, is represented by nanoemulsions, which in this case can be obtained using essential oil from *Artemisia* spp., knowing that the essential oil components have larvicidal activity. It has been demonstrated that the essential oil isolated from *A. vulgaris* has larvicidal and repellent action against *Aedes aegypti* through its major components (α-humulene, β-caryophyllene, and caryophyllene oxide) [206]. However, essential oils also have disadvantages such as high volatility [221,222], low water solubility [222,223], and lack of stability in the presence of air, heat, and light, followed by oxidation [223].

Based on the essential oil of *A. dracunculus* containing as major compounds p-allylanisole, cis- and beta-ocimene, limonene, and 3-methoxycinnamaldehyde, Osanloo et al. prepared, characterized, and tested 12 types of nanoemulsions with the same amount of essential oil (3.6 μL/mL). A final tested concentration of 18 ppm was obtained each time, but the concentration of tween 20 (Tw 20) with/without isopropyl alcohol (IPA) varied. After optimizing the synthesis by measuring median particle size, particle size distribution, and stability in undiluted and 1:200 diluted forms, two formulations were chosen for larvicidal studies against 3rd and 4th instar larvae of *Anopheles stephensi*. The first formula contained minimal amounts of surfactant/co-surfactant (2.5% Tw 20 and 2.5% IPA) with a median particle size of 15.6 nm, and the second formula contained only 10% Tw 20 (without IPA). After dilution, the second formula presented the smallest variation for median particle size (14.5 nm before, 11.20 nm after) and particle size distribution (1.30 nm before, 2.1 nm after). It is important to test formulations after dilution, given the fact that World Health Organization guidelines stipulate 1:100 or 1:200 dilution ratios for testing mosquito larvicides. The larvicidal effect of the two formulations was compared to that of the essential oil. Given the fact that the first formulation showed changes in the nanostructure after dilution, the recorded larvicidal effect was similar to that of the essential oil. However, for the second formula, the larvicidal effect was significantly higher. This can be explained by the stable formulation of the nanoemulsion after dilution and by the small size, which improves the ability of passing through the pores into the larva’s body [224].

In the quest of obtaining prolonged larvicidal activity and of overcoming the disadvantage of high volatility, Osanloo et al. continued the research by encapsulating the essential oil of *A. dracunculus* in chitosan–tripolyphosphate nanocapsules through the ion gelation technique. Briefly, several dilutions of the chitosan solution were added to a mixture containing different proportions of essential oil (0.36–1.6%), Tw 20 (2.5–3%), and ethanol (5.8–7.14%), and then, an aqueous solution of tripolyphosphate (TPP) of different concentrations was added. After determining the particle size and particle size distribution, the formulations with the smallest size (116–384 nm) were chosen to calculate the encapsulation efficiency and loading capacity. The encapsulation efficiency was found to be in the 25.10–39.66% range and the loading capacity was in the 14.88–22.24% range, the highest values being obtained for a nanocapsule size of 168 nm and a 1.6% essential oil, 0.8% chitosan, and 0.04% TPP content. It has been shown that after encapsulation, the essential oil had a sustained release, so the duration of action and the efficiency of larvicidal activity were higher (3–4 days) compared to that of the essential oil (1–2 days), which means that the volatile oil was protected from evaporation [221]. Another chitosan-based formulation, but with a concentration of 6.04% essential oil of *A. dracunculus* led to nanocapsules with 203 nm size, an encapsulation efficiency of 34.91%, and a larvicidal activity (against *Anopheles stephensi*), which was maintained for 10 days [225].

Essential oils are gaining more and more attention as pest control agents, given their high toxicity to stored grain insect pests, but low toxicity to humans and animals, with nanocapsule formulations overcoming some of their limitations [223]. Moreover, such formulations can offer a controlled release in a certain period of time [225], a more efficient use of the oil quantity by reducing the amount and frequency of administration, an an increase in stability, as well as environmentally friendly properties [222,226].

An example regarding this aspect is the testing of fumigant toxicity of nanocapsules obtained with *A. sieberi* essential oil by in situ polymerization against *Tribolium castaneum*, compared to the essential oil. Poly(urea-formaldehyde) was used as an external shell and the nanocapsules were spherical, with a diameter of approximately 80 nm. The results demonstrated a higher fumigant toxicity for nanocapsules against *Tribolium castaneum* after 7 days exposure time, as well as a higher persistence (half life time 28.73 days for nanocapsules and only 4.27 days for the essential oil) [222].

Another example in establishing the fumigant toxicity is that of nanocapsules prepared using *A. haussknechtii* essential oil by the interfacial compression polymerization method, which were tested against *Tribolium castaneum* and *Sitophilus oryza*. For the synthesis of nanocapsules, an optimization of the formula (emulsifier and co-emulsifier composition, temperature) was necessary. The results indicated an aggregation of nanocapsules when Tw 20 and Tw 40 were used as emulsifiers and poly vinyl pyrrolidone was used as co-emulsifier; meanwhile, using Tw 80 as emulsifier, at 45 °C for both micelle preparation and polymerization led to a good stability of nanocapsules with granular and spherical shape and a 40–50 nm size. The insecticidal activity varied depending on the species. Comparing the LC_50_ values for the fumigant toxicity of nanocapsules and of a reference product, a decrease of the concentration is observed in the case of nanocapsules. Stability testing has shown in the case of nanocapsules a constant release that was maintained for several days (up to 45 days) even if the mortality rate reached 50%. In contrast, the stability of the essential oil was comparatively lower [226]. 

Another environmentally friendly technique of formulating pesticides based on essential oils together with preventing their rapid evaporation is to incorporate them into solid lipid nanoparticles [227]. Lai et al. incorporated *A. arborescens* essential oil into solid lipid nanoparticles using Compritol 888 ATO as lipid and Poloxamer 188 or Miranol Ultra C32 as surfactants through the hot high-pressure homogenization technique. The obtained formulations demonstrated a good physical stability when stored for 2 months at different temperatures, and the in vitro testing showed a good capacity of reducing essential oil evaporation [213].

## 4. Conclusions

The paper reviewed recent articles on the biopesticide activity of *Artemisia* compounds and extracts. The ability of *Artemisia*-derived products to protect crops against fungi, bacteria, insects, nematodes, and weeds was analyzed. The vast majority of studies have been performed on plant extracts, especially volatile oils, and only a small number of articles have evaluated the properties of isolated compounds. The analysis of the literature data shows that the main substances with pesticide action in the genus *Artemisia* belong mainly to terpenoids (mono- and sesquiterpenes), but also to flavones, coumarins, and phenolic acids. Experiments show that the activity of the extract often exceeds that of the isolated compounds, and, in addition, the use of a mixture of substances prevents the appearance of the resistance of the pathogen to the pesticide used. Although of natural origin, *Artemisia* biopesticides are not without toxicity against non-target organisms and, to date, only a few investigations have been conducted into the environmental impact of these products. In addition, very few studies have evaluated the effectiveness of *Artemisia*-derived pesticides in the field, most being performed in vitro, and few in planta. The efficiency of these treatments in crops also depends on the mode of application, and the formulation of natural pesticides in modern and innovative structures such as nanosystems can improve their activity. The investigation of different possible alternatives to chemical pesticides could prove highly beneficial and, implicitly, the use of plants and nano-biopesticides can represent the future of research in this field. Plants can serve as good sources of compounds with such properties, while nano-sized formulations could provide fast, cost-effective synthesis methods and stability of formulation. At the same time, such formulations are bio-degradable, environmentally friendly, and provide an increased biological activity, as well as a slow release of active substances. Therefore, the application of nanosystems for the control of plant pathogens can be a rapidly emerging area in the management of plant diseases.

## Figures and Tables

**Table 1:** Antifungal activity of *Artemisia* extracts and compounds against phytopathogenic fungi.

*Artemisia* Species	Extract * or Compound Tested	Fungi	Inhibitory Dose	Type of Study	Reference
*A. abrotanum* fresh aerial parts	essential oil (eucalyptol)	*Sclerotinia sclerotiorum*	MIC = 1200 μL/L	in vitro	[17]
*A. absinthium* aerial parts	essential oil (cis-epoxyocimene, (−)-cis-chrysanthenol, chrysanthenyl acetate, linalool and β-caryophyllene)	*Botrytis cinerea*	ED_50_ = 0.01–0.07 mg/mL	in vitro	[18]
		*Fusarium moniliforme*	ED_50_ = 0.24–0.43 mg/mL		
		*F. oxysporum*	ED_50_ = 0.29–0.40 mg/mL		
		*F. solani*	ED_50_ = 0.24–0.50 mg/mL		
*A. absinthium* leaves	aqueous extract (1:1)	*Alternaria alternata*	79.75% inhibition	in vitro	[19]
		*Mucor piriformis*	73.04% inhibition		
		*Penicillium expansum*	75.42% inhibition		
*A. annua* fresh aerial parts	essential oil (artemisia ketone)	*Sclerotinia sclerotiorum*	MIC = 2400 μL/L	in vitro	[17]
*A. annua* aerial parts	essential oil (artemisia ketone, α-selinene and γ-terpineol)	*Alternaria solani*	EC_50_ = 21.78 mg/mL	in vitro agar diffusion	[20]
			EC_50_ = 14.18 mg/mL	in vitro spore germination	
*A. annua* leaves	methanol extract (ultrasound-assisted)	*Fusarium oxysporum*	36.94% inhibition	in vitro	[21]
	essential oil (camphor, germacrene D, β-caryophyllene, camphene)	*F. oxysporum*	MIC = 0.22 mg/mL		
		*F. solani*	MIC = 0.37 mg/mL		
	L-camphor	*F. oxysporum*	MIC = 0.11 mg/mL		
		*F. solani*	MIC = 0.31 mg/mL		
	DL-camphor	*F. oxysporum*	MIC = 0.14 mg/mL		
		*F. solani*	MIC = 0.16 mg/mL		
	β-caryophyllene	*F. oxysporum*	MIC = 0.13 mg/mL		
		*F. solani*	MIC = 0.23 mg/mL		
	camphene	*F. oxysporum*	MIC = 0.16 mg/mL		
		*F. solani*	MIC = 0.22 mg/mL		
	petroleum ether extract	*F. oxysporum*, *F. solani*	27.78% and 25% infection incidence, at 0.25 mg/g and 0.5 mg/g in the culture media, respectively	in vivo on *Panax notoginseng*	
*A. annua* whole plant	ethanol extract	*Aspergillus flavus*	14 mm inhibition zone at 200 μg/mL	in vitro	[22]
		*A. niger*	14.5 mm inhibition zone at 200 μg/mL		
*A. annua*	artemisinin	*Aspergillus fumigatus*	IC_50_ = 125 µg/mL IC_90_ = 250 µg/mL	in vitro	[23]
*A. arborescens*	essential oil (chamazulene, camphor)	*Rhizoctonia solani*	47.2% inhibition at 12.5 µL/20 mL medium 100% inhibition at 50 µL/20 mL medium	in vitro	[24]
*A. argyi* leaves	essential oil (caryophyllene oxide, neointermedeol, borneol, α-thujone, β-caryophyllene)	*Aspergillus niger*	MIC = 6.25 µL/mL	in vitro	[25]
*A. argyi* inflorescence	essential oil (spathulenol, juniper camphor, caryophyllene oxide, terpineol, 1,8-cineole, borneol, camphor, chamazulene)	*Alternaria alternata*	84.7% inhibition at 1000 mg/L	in vitro	[26]
		*Botrytis cinerea*	93.3% inhibition at 1000 mg/L		
*A. austriaca* fresh aerial parts	essential oil (camphor)	*Sclerotinia sclerotiorum*	MIC = 2400 μL/L	in vitro	[17]
*A. caerulescens* ssp. *densiflora*	essential oil (terpinen-4-ol, p-cymene, γ-terpinene, 1,8-cyneole, α-terpineol)	*Alternaria* spp.	20 mm inhibition zone at 1:2 dilution	in vitro	[27]
		*Aspergillus* spp.	12 mm inhibition zone at 1:1 dilution		
		*Fusarium* spp.	16 mm inhibition zone at 1:8 dilution		
*A. campestris* aerial parts	methanol extracts (1:10)	*Aspergillus niger*	32.5–33.1 mm inhibition zone at 20 µg/mL	in vitro	[28]
*A. campestris* aerial parts	essential oil (α-pinene, β-pinene, β-myrcene, germacrene D)	*Aspergillus flavus*	MIC = 2.5 μL/mL MFC = 2.5 μL/mL	in vitro	[29]
		*Aspergillus niger*	MIC = 10 μL/mL MFC >20 μL/mL		
		*Aspergillus ochraceus*	MIC = 2.5 μL/mL MFC = 5 μL/mL		
		*Aspergillus parasiticus*	MIC = 2.5 μL/mL MFC = 5 μL/mL		
		*Fusarium culmorum*	MIC = 2.5 μL/mL MFC = 5 μL/mL		
		*Fusarium graminearum*	MIC = 1.25 μL/mL MFC = 1.25 μL/mL		
		*Fusarium moniliforme*	MIC = 2.5 μL/mL MFC = 2.5 μL/mL		
		*Penicillium citrinum*	MIC = 5 μL/mL MFC > 20 μL/mL		
		*Penicillium expansum*	MIC = 2.5 μL/mL MFC = 2.5 μL/mL		
		*Penicillium viridicatum*	MIC = 10 μL/mL MFC > 20 μL/mL		
*A. chamaemelifolia* aerial parts	essential oil (carvacrol, thymol, p-cymene α-cadinol)	*Aspergillus oryzae*	MIC = 312.5 μg/mL MFC = 312.5 μg/mL	in vitro	[30]
		*Aspergillus niger*	MIC = 2500 μg/mL MFC = 2500 μg/mL		
		*Byssochlamys spectabilis*	MIC = 625 μg/mL MFC = 625 μg/mL		
		*Paecilomyces variotii*	MIC = 625 μg/mL MFC = 625 μg/mL		
		*Penicillium chrysogenum*	MIC = 625 μg/mL MFC = 625 μg/mL		
		*Trichoderma harizanum*	MIC = 312.5 μg/mL MFC = 312.5 μg/mL		
*A. dracunculus* fresh aerial parts	essential oil (sabinene)	*Sclerotinia sclerotiorum*	MIC = 2400 μL/L	in vitro	[17]
*A. dracunculus* var. *pilosa* fresh aerial parts	essential oil (borneol)		MIC = 2400 μL/L		
*A. herba-alba* aerial parts	essential oil (davanone, camphor, thujone)	*Fusarium moniliforme*	MIC = 0.5%	in vitro direct contact	[31]
		*Fusarium oxysporum*	MIC = 0.5%		
		*Fusarium solani*	MIC = 0.75%		
		*Stemphylium solani*	MIC = 0.75%		
*A. herba-alba* leaves	essential oil (β-thujone, α-thujone camphor)	*Penicillium aurantiogriseum*	100% inhibition at 0.89%	in vitro	[32]
		*P. viridicatum*	100% inhibition at 1.33%		
*A. herba-alba* fresh leaves	essential oil	*Mucor rouxii*	100% inhibition at 1000 µg/mL	in vitro	[33]
		*Penicillium citrinum*	100% inhibition at 1000 µg/mL		
	carvone	*Mucor rouxii*	IC_50_ = 7 µg/mL		
		*Penicillium citrinum*	IC_50_ = 5 µg/mL		
	piperitone	*Mucor rouxii*	IC_50_ = 1.5 µg/mL		
		*Penicillium citrinum*	IC_50_ = 2 µg/mL		
*A. herba-alba* aerial parts	chloroform-methanol extract	*Fusarium solani*	MIC = 62.5 μg/disc	in vitro	[34]
	11-epiartapshin		MIC = 50 μg/disc		
*A. incisa* aerial parts	santolinylol-3-acetate	*Aspergillus flavus*	MIC = 300 μg/mL	in vitro	[35]
	santolinylol		MIC = 300 μg/mL		
	*trans*-ethyl cinnamate		MIC = 500 μg/mL		
	isofraxidin		MIC = 400 μg/mL		
	eupatorin		MIC = 1000 μg/mL		
	scopoletin		inactive		
	esculetin		inactive		
*A. judaica* aerial parts	essential oil (piperitone, 3-bornanone)	*Aspergillus niger*	MIC = 1.25 μg/disc	in vitro	[36]
		*Fusarium solani*	MIC = 2.5 μg/disc		
*A. khorasanica* aerial parts	essential oil (davanone, p-cymene, Z-citral, β-ascaridol, thymol)	*Fusarium moniliforme*	MIC = 2000 µL/L	in vitro	[37]
		*Fusarium solani*	MIC = 1500 µL/L		
		*Rhizoctonia solani*	MIC = 1000 µL/L		
		*Tiarosporella phaseolina*	MIC = 2000 µL/L		
*A. lavandulaefolia* aerial parts	essential oil (eucalyptol, (-)-terpinen-4-ol, α-terpineol)	*Alternaria solani*	EC_50_ = 10.45 mg/mL	in vitro agar diffusion	[20]
			EC_50_ = 6.64 mg/mL	in vitro spore germination	
*A. lerchiana* fresh aerial parts	essential oil (eucalyptol)	*Sclerotinia sclerotiorum*	MIC = 2400 μL/L	in vitro	[17]
*A. maritima* aerial parts	essential oil (1,8-cineole, chrysanthenone, germacrene D, borneol)	*Aspergillus flavus*	35.4% inhibition at 10 µL/plate	in vitro	[38]
		*A. niger*	60.6% inhibition at 10 µL/plate		
		*A. ochraceus*	56.1% inhibition at 10 µL/plate		
		*A. parasiticus*	32.45% inhibition at 10 µL/plate		
		*A. terreus*	58.3% inhibition at 10 µL/plate		
		*Fusarium moniliforme*	33.9% inhibition at 10 µL/plate		
		*Penicillium chrysogenum*	28.6% inhibition at 10 µL/plate		
*A. nilagirica* shoot	essential oil (camphor, β-caryophyllene, α-thujone, sabinene)	*Aspergillus flavus, A. niger*, *A. ochraceus*	MIC = 0.29 μL/mL MFC = 0.58 μL/mL	in vitro	[39]
			100% mycotoxin inhibition at 0.16 μL/mL		
		*Aspergillus terreus, Cladosporium cladosporioides, Fusarium moniliforme, Fusarium oxysporum, Mucor mucedo, Penicillium expansum, P. funiculosum, Rhizopus stolonifer*	100% inhibition at 0.29–0.58 μL/mL	in vitro	
			0% disease incidence at 300 μL/2 L	in situ fumigation test on grapes, 10 days storage	
*A. nilagirica* aerial parts	essential oil (1,5-heptadiene-4-one,3,3,6-trimethyl, artemisia alcohol, α-ionone, benzene, methyl (1-methylethyl))	*Aspergillus flavus* toxigenic strain	MIC = 1.4 µL/mL MFC = 4.0 µL/mL	in vitro	[40]
		*Alternaria alternata, Aspergillus flavus, A. minutus, A. niger, A. sydowii, A. terreus, Cheatomium spirale, Curvularia lunata, Mucor* spp., *Mycelia sterilia Penicillium italicum, P. purpurogenum, Rhizopus stolonifer*,	70–100% inhibition at 1.4 µL/mL	in vitro	
			71% protection from fungal contamination at 1.4 μL/mL in air	in situ on *Eleusine* *coracana* seeds, 12 months storage	
*A. nilagirica* aerial parts	essential oil (α-thujone, β-thujone, germacrene D, 4-terpineol, β-caryophyllene, camphene, borneol)	*Macrophomina phaseolina*	ED_50_ = 93.23 mg/L	in vitro	[41]
		*Rhizoctonia solani*	ED_50_ = 85.75 mg/L		
		*Sclerotium rolfsii*	ED_50_ = 87.63 mg/L		
*A. nilagirica* leaves	essential oil (α-thujone, borneol, β-thujone, 1,8-cineole)	*Phytophthora capsici*	100% inhibition at 100 ppm	in vitro	[42]
*A. pallens* leaves	methanol extract 1:10	*Sclerospora graminicola*	Inhibition of zoosporangium formation	in vitro	[43]
*A. parviflora* twigs	methanol extract 1:1	*Sclerospora graminicola*	Inhibition of zoosporangium formation		
*A. pontica* fresh aerial parts	essential oil (eucalyptol)	*Sclerotinia sclerotiorum*	MIC = 2400 μL/L	in vitro	[17]
*A. proceriformis* fresh leaves	essential oil (α-thujone)	*Aspergillus carbonarius*	MIC = 10.6 mg/mL	in vitro	[44]
		*Aspergillus niger*	MIC = 21.2 mg/mL		
		*Fusarium graminearum*	MIC = 10.6 mg/mL		
		*F. verticillioides*	MIC = 10.6 mg/mL		
		*Septoria glycines *	MIC = 2.7 mg/mL		
		*Septoria tritici*	MIC = 2.7 mg/mL		
*A. santonica* fresh aerial parts	essential oil (α-thujone)	*Sclerotinia sclerotiorum*	MIC = 2400 μL/L	in vitro	[17]
*A. scoparia* aerial parts	essential oil (acenaphthene, curcumene, (+) caryophyllene oxide, spathulenol, methyl eugenol, β-caryophyllene)	*Alternaria solani*	EC_50_ = 12.2 mg/mL	in vitro agar diffusion	[20]
			EC_50_ = 3.8 mg/mL	in vitro spore germination	
*A. sieberi* aerial parts	1*R*, 8*S*-dihydroxy- 11*R*,13-dihydrobalchanin	*Fusarium solani*	6 mm inhibition zone at 200 μg/10 μL	in vitro	[45]
	11-epiartapshin		7 mm inhibition zone at 200 μg/10 μL		
	3′-hydroxygenkwanin		8 mm inhibition zone at 200 μg/10 μL		
*A. sieberi* aerial parts	essential oil (camphor, 1,8-cineole, camphene, chrysanthenone)	*Botrytis cinerea*	100% inhibition at 1000 µl/L	in vitro	[46]
*A. stricta* f. *stricta* aerial parts	essential oil (capillene, spathulenol, β-caryophyllene)	*Aspergillus flavus, Aspergillus niger, Sporothrix schenckii*	MIC = 0.625 mg/mL	in vitro	[47]
*A. terrae-albae leaves*	camphor, 1,8-cineole, camphene, β-thujone	*Aspergillus carbonarius*	MIC > 1.20 mg/mL	in vitro	[48]
		*Aspergillus niger*	MIC > 1.20 mg/mL		
		*Fusarium graminearum*	MIC = 0.60–1.20 mg/mL		
		*Fusarium verticillioides*	MIC = 0.60 mg/mL		
*A. turanica* aerial parts	essential oil (1,8-cineol, cis-verbenyl acetate, camphor)	*Aspergillus niger*	68.6% inhibition at 1 μL/mL	in vitro	[49]
*A. vulgaris* whole plant	crude methanol extract (1:10)	*Botrytis cinerea*	60% inhibition at 2 mg/mL	in vivo on *Cucumis sativus*	[50]
		*Blumeria graminis* f. sp. *hordei*	25% inhibition at 2 mg/mL	in vivo on *Hordeum sativum*	
		*Magnaporthe grisea*	16% inhibition at 2 mg/mL	in vivo on *Oryza sativa*	
		*Phytophthora infestans*	32% inhibition at 2 mg/mL	in vivo on *Lycopersicon esculentum*	
		*Puccinia recondita*	52% inhibition at 2 mg/mL	in vivo on *Triticum aestivum*	
		*Thanatephorus cucumeris*	9.3% inhibition at 2 mg/mL	in vivo on *Oryza sativa*	
*A. vulgaris* leaves	methanol extract 1:1	*Sclerospora graminicola*	Inhibition of zoosporangium formation	in vitro	[43]
*A. vulgaris* fresh aerial parts	essential oil (germacrene D)	*Sclerotinia sclerotiorum*	MIC = 2400 μL/L	in vitro	[17]

* To highlight the active compounds, the major constituents of the volatile oils were noted in parentheses.

**Table 2:** Insecticidal activity of *Artemisia* compounds and extracts.

*Artemisia* spp.	Extract or Compound Tested	Target Species	Reference
*A. absinthium*	essential oil	*Leptinotarsa decemlineata* *Myzus persicae* *Rhopalosiphum padi* *Spodoptera littoralis*	[18]
	essential oil	*Trialeurodes vaporariorum* *Tuta absoluta*	[66]
	essential oil	*Tetranychus cinnabarinus*	[67]
	essential oil	*Diaphania hyalinata*	[68]
	methanol extract	*Sitophilus oryzae*	[69]
	essential oil	*Orysaephilus surinamensis* *Tribolium castaneum*	[70]
	powdered plant	*Oryzaephilus surinamensis*	[71]
	water extract ethanol extract	*Hyphantria cunea*	[72]
	supercritical extracts	*Spodoptera littoralis*	[73]
	essential oil	*Myzus persicae*	[74]
	essential oil carvacrol (−)-α-bisabolol chamazulene	*Diaphorina citri*	[75]
*A. annua*	methanol extract essential oil	*Helicoverpa armigera*	[76]
	methanol extract artemisinic acid artemisinin scopoletin arteannuin-B deoxy-artemisinin artemetin casticin chrysosplenetin	*Helicoverpa armigera*	[77]
	essential oil	*Glyphodes pyloalis*	[78]
	methanol extract	*Pieris rapae*	[79]
	methanol extract	*Hyphantria cunea*	[80]
	methanol extract	*Glyphodes pyloalis*	[81]
	essential oil	*Diaphania hyalinata*	[68]
*A. arborescens*	essential oil	*Rhysopertha dominica*	[24]
*A. argyi*	ethanol extract	*Brevicoryne brassicae*	[82]
	essential oil	*Diaphania hyalinata*	[68]
	water extract ethanol extract	*Hyphantria cunea*	[72]
	essential oil	*Plodia interpunctella*	[83]
*A. frigida*	essential oil	*Liposcelis bostrychophila* *Sitophilus zeamais*	[84]
	essential oil terpinen-4-ol verbenone camphene α-terpineol α-terpinyl acetate	*Lasioderma serricorne* *Liposcelis bostrychophila* *Tribolium castaneum*	[85]
*A. herba-alba*	essential oil	*Orysaephilus surinamensis* *Tribolium castaneum*	[70]
*A. judaica*	essential oil	*Sitophilus orizae*	[64]
*A. lavandulaefolia*	essential oil 1,8-cineole chamazulene β-caryophyllene	*Lasioderma serricorne*	[86]
*A. monosperma*	essential oil	*Sitophilus orizae*	[64]
	essential oil	*Aphis nerii*	[87]
*A. nilagirica*	cow urine extract	*Scirpophaga incertulas*	[88]
*A. spicigera*	essential oil	*Dendroctonus micans*	[89]
*A. vulgaris*	essential oil	*Callosobruchus maculatus* *Rhyzopertha dominica* *Tribolium castaneum*	[90]
	essential oil	*Diaphania hyalinata*	[68]
	water extract ethanol extract	*Hyphantria cunea*	[72]

**Table 3:** Phytotoxic activity of *Artemisia* compounds and extracts.

*Artemisia* Species	Extract * or Compound Tested	Weed/Target Plant	Observed Effect	Reference
*A. absinthium* aerial parts	essential oil (cis-epoxyocimene, (−)-cis-chrysanthenol, chrysanthenyl acetate, linalool and β-caryophyllene)	*Lolium perene*	Suppression of root and leaf growth No effect on seed germination	[18]
		*Lactuca sativa*	Suppression of root and leaf growth No effect on seed germination	
*A. absinthium* fresh aerial parts	essential oil (β-thujone, chamazulene)	*Sinapis arvensis*	Complete inhibition of seed germination and seedling growth at 2 µL/mL	[114]
*A. absinthium* leaves	aqueous extract 1:10 *w*/*v*	*Parthenium hysterophorus*	Inhibition of seed germination, shoot and root growth, reduction of chlorophyll and carotenoid content, at 25, 50, 75, and 100% Enhanced malondialdehyde levels, phenolic content and increased activity of antioxidative enzymes, at 25, 50, 75, and 100%	[105]
*A. absinthium* shoot and root	aqueous extract	*Chenopodium album*	Decreases growth criteria (root and shoot length and fresh weight, number of leaves) at 1–100 mg/mL No effect on seed germination Increased peroxidase and superoxide dismutase activity in root	[115]
*A. afra* leaves	aqueous extract	*Triticum aestivum*	No effect on seed germination	[116]
		*Brassica napus*	Complete inhibition of seed germination	
		*Medicago sativa*	Increased germination rate	
		resistant and non-resistant *Lolium* spp.	Significant inhibition of seed germination	
*A. annua* flower heads	essential oil (1,8-cineole, trans-sabinyl acetate, artemisia ketone, camphor α-pinene)	*Amaranthus retroflexus*	In vitro, complete inhibition of seed germination, at 10 and 100 µg/L In vivo, plant death, at the cotyledon stage (100 mg/L) and true leaf stage (1000 mg/L)	[117]
		*Setaria viridis*	In vitro, complete inhibition of seed germination, at 100 µg/L In vivo, plant death, at the cotyledon stage (100 mg/L) and true leaf stage (1000 mg/L)	
*A. annua* aerial parts	artemisinin arteannuin B artemisinic acid	*Secale cereale, Hordeum vulgare, Artemisia annua, Portulaca oleracea, Amaranthus blitun, Lactuca sativa, Raphanus sativus*	Inhibition of seed germination Inhibition of shoot and root growth	[118]
*A. annua*	artemisinin	*Lactuca sativa*	Inhibition of root and shoot elongation, reduced cell division and cell viability in root tips, at 10 µM Reduced chlorophyll a and b levels Increased malondialdehyde and proline levels, at 1 µM	[119]
*A. annua*	artemisinin	*Arabidopsis thaliana*	Reduction of fresh biomass, chlorophyll a, b, and leaf mineral contents at 40–160 μM Reduction of photosynthetic efficiency, yield, and electron transport rate, calcium and nitrogen levels at 80 and 160 μM Elevated lipid peroxidation (malondialdehyde contents) at 80 and 160 μM	[120]
*A. arborescens* shoot	sesamin ashantin	*Agrostis stolonifera*, *Lactuca sativa*	Growth inhibition at 1 mg/mL	[107]
	sesamin	*Lemna paucicostata*	Growth inhibition IC_50_ = 401 μM	
	ashantin	*Lemna paucicostata*	Growth inhibition IC_50_ = 224 μM	
*A. arborescens* leaf litter	crude methanol extract	*Lactuca sativa, Raphanus sativus, Amaranthus retroflexus, Cynodon dactylon*	Inhibition of seed germination ED_50_ = 1.61–3.05 mg/mL Inhibition of root growth ED_50_ = 1.22–3.14 mg/mL	[121]
	hexane, chloroform, and ethyl acetate fractions		Inhibition of seed germination ED_50_ = 1.19–6.25 mg/mL Inhibition of root growth ED_50_ = 0.92–3.98 mg/mL	
*A. arborescens* aerial part	crude methanol and aqueous extracts	*Lactuca sativa*	Inhibition of seed germination and root growth ED_50_ = 0.5–2.8 mg/mL	[122]
	ethyl acetate, n-hexane, chloroform, n-butanol fractions		Inhibition of seed germination and root growth ED_50_ = 0.4–5.4 mg/mL	
*A. argyi* leaves	water extract (caffeic acid, schaftoside, 4-caffeoylquinic acid, 5-caffeoylquinic acid, 3,5-dicaffeoylquinic acid and 3-caffeoylquinic acid)	*Brassica pekinensis, Lactuca sativa, Oryza sativa*	Inhibition of germination, root and stem growth, and biomass (at 50, 100, and 150 ng/mL)	[108]
		*Brassica pekinensis, Lactuca sativa, Oryza sativa, Portulaca oleracea, Oxalis corniculata, Setaria viridis*	Inhibition of germination and growth in pot experiment (*A. argyi* powder mixed into sand soil at the ratio 100:0, 100:2, 100:4, and 100:8)	
*A. campestris* leaves	essential oil (β-pinene, 1, 8-cineole, p-cymene, myrcene)	*Daucus carota, Cicer arietinum, Phaseolus vulgaris, Triticum sativum*	Reduces seed germination at 1000–2000 ppm Enhances seed germination at 100 ppm Delays the germination of *D. carota* seeds	[123]
*A. dracunculus* aerial parts	essential oil	*Medicago minima, Rumex crispus, Taraxacum officinale*	No effect on seed germination at 0.3–1.2 mg/L	[124]
*A. dracunculus*	leachate	*Lactuca sativa*	Radicle growth inhibition	[125]
*A. fragrans* aerial parts	essential oil (α-thujone, camphor, 1,8-cineole, β-thujone)	*Convolvulus arvensis*	Important reduction in the shoot, root, and plant length, shoot and root fresh weight, shoot and root dry weight Inhibited seed germination Significant decrease of photosynthetic pigments and antioxidant enzymes Increased production of H_2_O_2_ and malondialdehyde content, and membrane leakage	[126]
*A. fragrans* roots, leaves, and flowers	methanol extracts	*Raphanus raphanistrum*	Inhibition of root growth at 1000 ppm Inhibition of seed germination at 7500 ppm	[56]
*A. frigida*	volatile organic compounds (1,8-cineole, camphene, (E)-3-hexen-1-ol acetate, α-terpineol, β-terpineol)	*Melitotus suaveolens, Sorghum sudanense, Elymus dahuricus, Agropyron cristatum*	Significantly decreases the seed germination and seedling growth	[127]
*A. judaica* aerial parts	essential oil (piperitone, 3-bornanone)	*Lactuca sativa*	Reduced seed germination, shoot and root growth at 250–1000 µL/L	[36]
*A. lavandulaefolia* leaves	aqueous extract	*Lactuca sativa, Artemisia princeps, Achyranthes japonica, Oenothera odorata, Plantago asiatica, Aster yomena, Elsholzia ciliata, Raphanus sativus*	Inhibition of root growth Inhibition of seed germination	[128]
	essential oil (1,8-cineole, α-terpineol, α-terpinene, camphor, azulene, 2-buten-1-ol)			
*A. monosperma* aerial parts	aqueous extract	*Phaseolus vulgaris*	Stimulation of seed germination at 1% and 2% concentration Inhibition of seed germination at 3% and 4% concentration Inhibition of amylase and protease activity	[129]
*A. monosperma* aerial parts	aqueous extract	*Medicago polymorpha*	Reduction of germination percentage, plumule and radicle growth, and seedling dry weight	[130]
	crude plant powder mixed with clay loam soil		Inhibitory effects on leaf area index, total photosynthetic pigments, total available carbohydrates and total protein, in pot culture bioassay	
*A. scoparia* fresh leaves	essential oil (β-myrcene, (+)-limonene, (Z)-β-ocimene, γ-terpinene)	*Avena fatua, Cyperus rotundus, Phalaris minor*	Important reduction in germination, seedling growth, and dry matter at 0.07–0.7 mg/mL	[131]
*A. scoparia* fresh leaves	essential oil (p-cymene, β-myrcene, (+)-limonene)	*Achyranthes aspera, Cassia occidentalis, Parthenium hysterophorus, Echinochloa crus-galli, Ageratum conyzoides*	Inhibition of seed germination, root and shoot growth at 10, 25, and 50 µg oil/g sand Chlorosis, necrosis and complete wilting of plants 1 to 7-days after spraying with oil (2%, 4%, and 6%, *v*/*v*) Significant decline in chlorophyll content and cellular respiration, electrolyte leakage	[132]
*A. sieversiana* fresh aerial parts	essential oil (α-thujone, eucalyptol)	*Amaranthus retroflexus, Medicago sativa, Poa annua, Pennisetum alopecuroides*	Inhibition of root and shoot growth IC_50_ = 1.89–4.69 mg/mL	[133]
	α-thujone		IC_50_ = 1.55–6.21 mg/mL	
	eucalyptol		IC_50_ = 1.42–17.81 mg/mL	
	α-thujone and eucalyptol mixture		IC_50_ = 0.23–1.05 mg/mL	
*A. terrae-albae* aerial parts	essential oil (α-thujone, β- thujone, eucalyptol, camphor)	*Amaranthus retroflexus*	Reduces root and shoot growth at 1.5 μg/mL Completely inhibits seed germination at 3 μg/mL	[134]
		*Poa annua*	Reduces root and shoot growth at 1.5 μg/mL Completely inhibits seed germination at 5 μg/mL	
*A. verlotiorum* flower heads	essential oil (chrysanthenone, 1,8-cineole, β-pinene, camphor 2,6-dimethyl phenol, β-caryophyllene)	*Amaranthus retroflexus*	In vitro, complete inhibition of seed germination, at 10 and 100 µg/L In vivo, plant death, at the cotyledon stage (100 mg/L) and true leaf stage (1000 mg/L)	[117]
		*Setaria viridis*	In vitro, inhibition of seed germination, at 10 and 100 µg/L In vivo, plant death, at the cotyledon stage (1000 mg/L) and true leaf stage (1000 mg/L)	
*A. vulgaris* aerial parts	aqueous extract	*Amaranthus retroflexus*	Inhibition of seed germination, radicle, and hypocotyl length at 7.5% to 10% *w*/*v*, in Petri dish bioassays Inhibition of seedling emergence and plant growth, in pot culture bioassays	[104]
		*Zea mays*	Stimulation of radicle and mesocotyl growth at 7.5% to 10% *w*/*v*, in Petri dish bioassays Stimulation of plant biomass, in pot culture bioassays	
*A. vulgaris* leaves and flowers	essential oil	*Agrostemma githago, Amaranthus retroflexus, Cardaria draba, Chenopodium album, Echinochloa crus-galli, Reseda lutea, Rumex crispus, Trifolium pratense*	Inhibition of root and shoot growth and reduction of germination rate (at 2, 5, 10 and 20 μL/plate)	[135]
*A. vulgaris* root	aqueous extracts	*Triticum aestivum* (winter wheat)	Inhibition of shoot and root growth by all concentrations (1:6250 to 1:10)	[136]
		*Brassica napus* spp. *oleifera* var. *biennis* (winter oilseed rape)	Significant inhibition of germination at the 1:10 concentration	
*A. vulgaris* aerial parts			Significant inhibition of root growth at 1:10 concentration Stimulation of shoot growth	

* To highlight the active compounds, the major constituents of the volatile oils were noted in parentheses.
